# Lessons from Nature: Advances and Perspectives in Bionic Microwave Absorption Materials

**DOI:** 10.1007/s40820-024-01591-2

**Published:** 2024-12-30

**Authors:** Dashuang Wang, Tuo Ping, Zhilan Du, Xiaoying Liu, Yuxin Zhang

**Affiliations:** 1 College of Materials Science and Engineering, Chongqing University, Chongqing, 400044 People’s Republic of China; 2 Army Logistics Academy of PLA, Chongqing, 401331 People’s Republic of China; 3 Beijing Spacecrafts, China Academy of Space Technology, Beijing, 100194 People’s Republic of China

**Keywords:** Bionic, Structural design, Microwave absorption, Electromagnetic theory

## Abstract

Inspired by the remarkable electromagnetic response capabilities of the complex morphologies and subtle microstructures evolved by natural organisms, this paper delves into the research advancements and future application potential of bionic microwave-absorbing materials (BMAMs). It outlines the significance of achieving high-performance microwave-absorbing materials through ingenious microstructural design and judicious composition selection, while emphasizing the innovative strategies offered by bionic manufacturing. Furthermore, this work meticulously analyzes how inspiration can be drawn from the intricate structures of marine organisms, plants, animals, and non-metallic minerals in nature to devise and develop BMAMs with superior electromagnetic wave absorption properties. Additionally, the paper provides an in-depth exploration of the theoretical underpinnings of BMAMs, particularly the latest breakthroughs in broadband absorption. By incorporating advanced methodologies such as simulation modeling and bionic gradient design, we unravel the scientific principles governing the microwave absorption mechanisms of BMAMs, thereby furnishing a solid theoretical foundation for understanding and optimizing their performance. Ultimately, this review aims to offer valuable insights and inspiration to researchers in related fields, fostering the collective advancement of research on BMAMs.

## Highlights

- This review describes the classification of bionic objects of bionic wave-absorbing materials in detail. From marine organisms, insects, plants to animals, different bionic objects will bring diversified influences and applications.
- The multifunctional applications of bionic microwave absorption materials are systematically introduced in this paper, from microwave absorption to anti-corrosion, to mechanics, electronics, wearable devices, etc.
- The theoretical basis and simulation calculation of bionic microwave absorption materials are also discussed.

## Graphical abstract

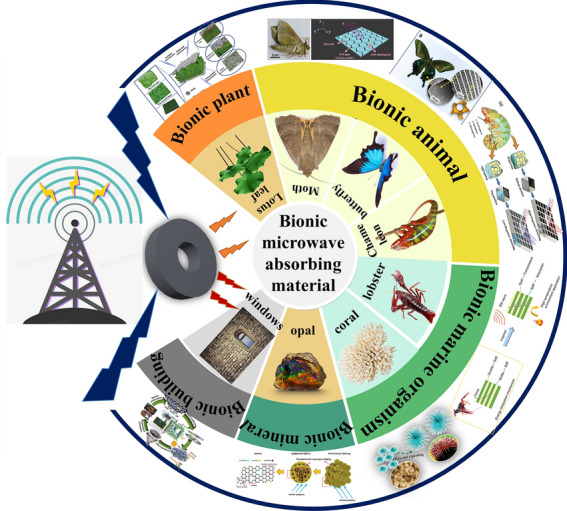

## Introduction

In recent years, with the continuous development of microwave heating, radar, and aerospace, people have paid more and more attention to microwave-absorbing materials (MAMs), and their development and application are increasingly extensive. In civil use, microwave is widely used in communication, radar detection and other fields [1, 2]. This not only provides convenience for human activities, but also leads to serious electromagnetic wave absorption (EMA) pollution and electromagnetic interference [3, 4]. In the military, microwave radar has been widely used in various countries and has become a ubiquitous anti-stealth technology, which has become an important issue related to national security [5, 6]. Therefore, researchers all over the world have devoted themselves to studying new MAMs, hoping to effectively absorb EWA to solve the above problems.

**Fig. 1:**
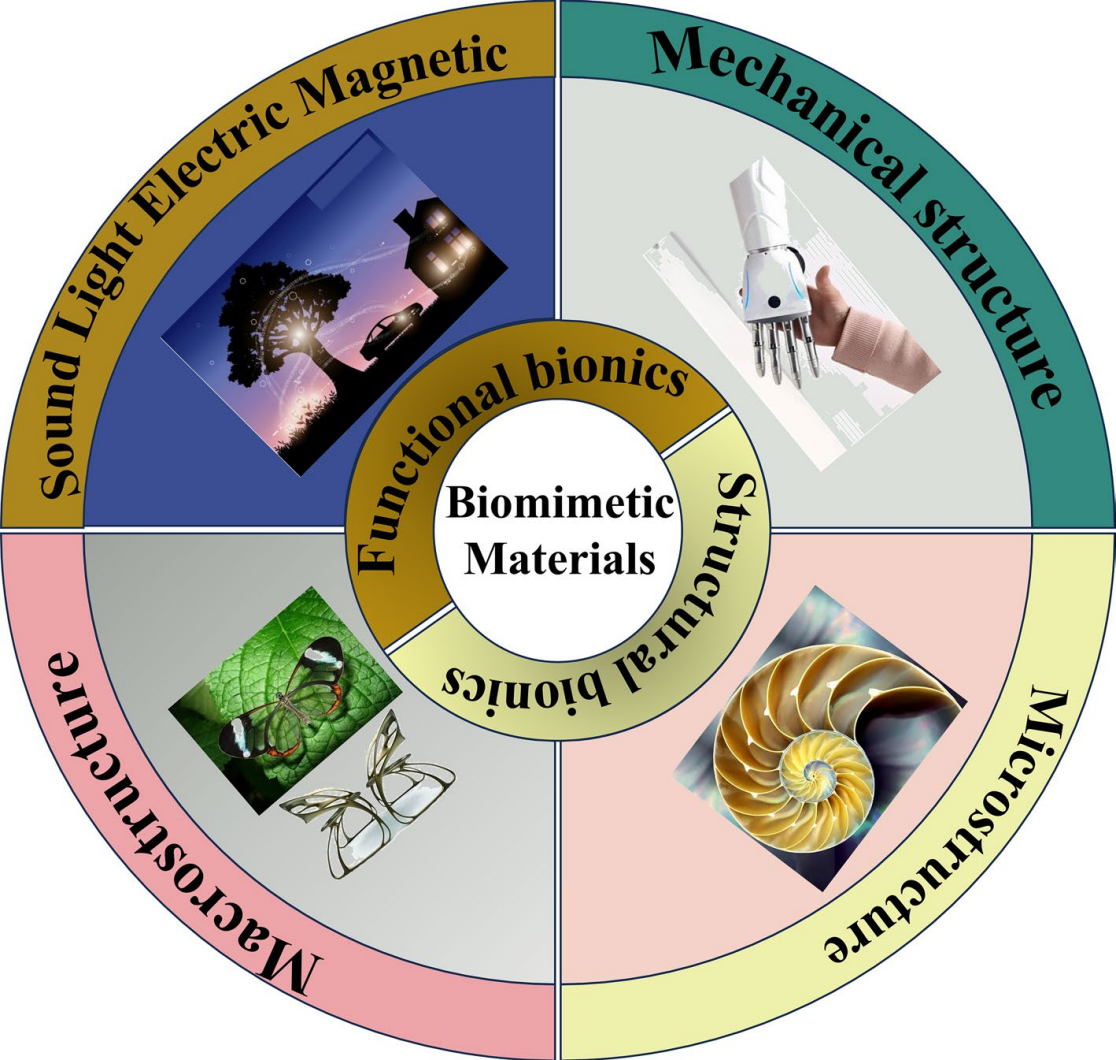
Biomimetic mechanism of biomimetic materials

Bionics, a field that emulates biological principles in designing technical systems, aims to endow artificial systems with similar or even superior biological functions [7, 8]. Through advancements in microscopic technologies, it has been revealed that organisms, visually appearing “plain” yet possessing remarkable functionalities, possess intricate microstructures. These functionalities do not solely stem from atomic or molecular arrangements but rather from the sequential assembly of “functional primitives,” components several orders of magnitude larger than molecules and atoms [9–11]. As depicted in Fig. 1, the objects of bionic inspiration encompass diverse living organisms, ranging from animals and plants to human organs [12]. Bionics achieves its objectives through two primary aspects: structural bionics and functional bionics. Structural bionics involves replicating the macroscopic or microscopic architectures of organisms to serve unexpected purposes[13]. Meanwhile, functional bionics mimics the mechanical, optical, acoustic, electrical, and magnetic capabilities inherent in organisms. For instance, the micro-nanohierarchical “papillae” structures on lotus leaves, composed of waxy materials, enable super-hydrophobic and self-cleaning properties [14]. Additionally, the periodic arrangement of guanine particles on chameleons’ bodies forms natural photonic crystals, exhibiting a dynamic range of colors [15], illustrating the richness and complexity of functional biomimicry. Furthermore, it is worth noting that chemical composition also plays a pivotal role in bionics, as it often dictates the unique properties and functionalities of biological structures. By understanding and incorporating the chemical composition of natural materials, researchers can develop artificial systems with enhanced performance and novel functionalities [16].

**Fig. 2:**
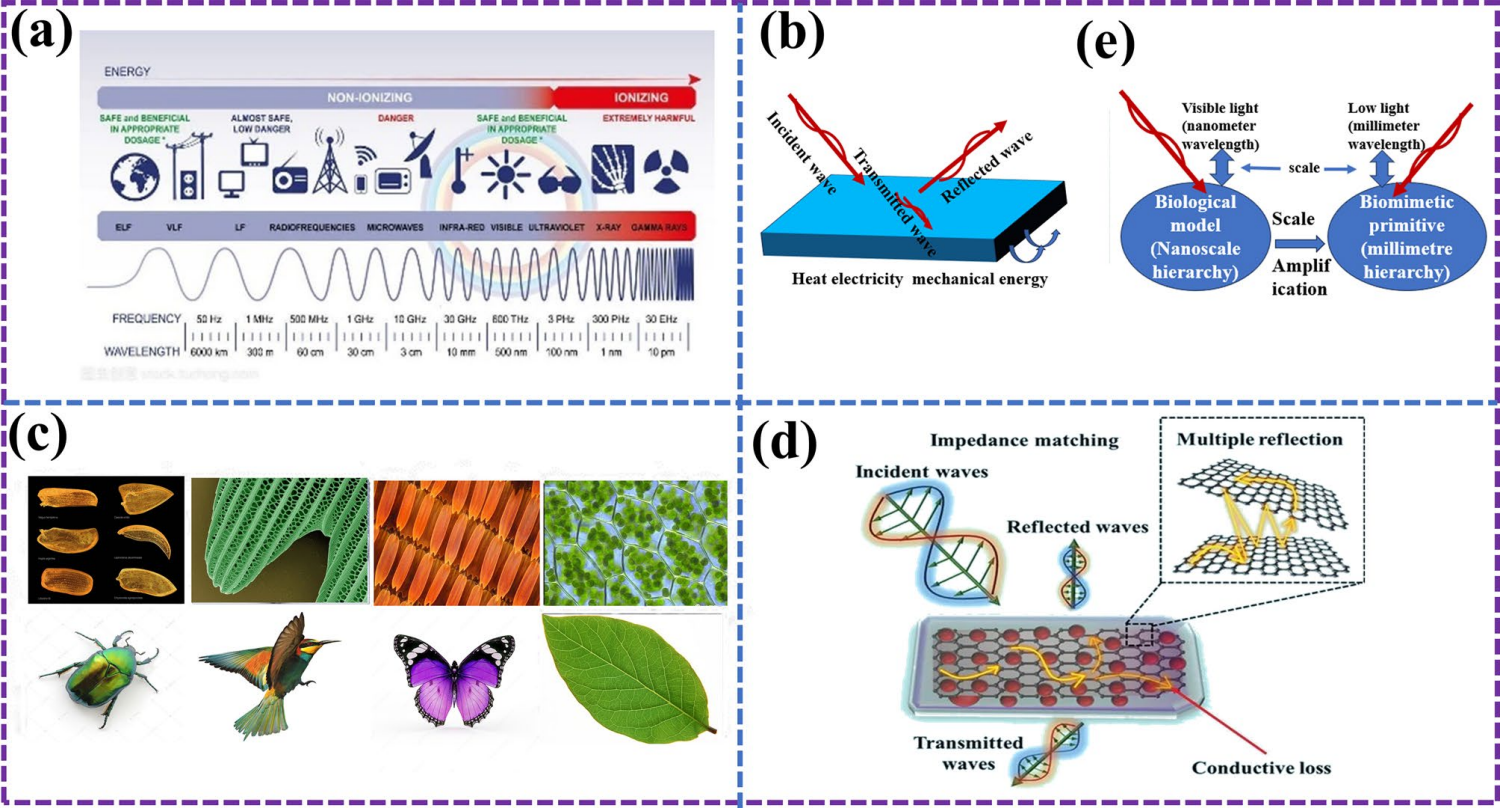
**a** Frequencies, wavelengths corresponding to different bands and their applications [17]; **b** absorption mechanism of MAMs [23]; **c** different biological surface functional primitives and their order forms; **d** schematic diagram of bionics combined with MAMs [28, 29]; **e** schematic diagram of bionic primitive scale design [30]

As shown in Fig. 2a [17, 18], if the frequencies of each band are arranged from low to high, they are radio wave, microwave, infrared ray, visible light, ultraviolet ray, X-ray, and gamma ray, respectively. EMAs with a frequency of 300 MHz–300 GHz are called microwaves, which are in the high frequency band of radio waves [19, 20]. According to the absorption mechanism, the absorbers of MAMs are mainly divided into dielectric type, magnetic medium type, and resistance type [21, 22]. The working principle of the MAMs is shown in Fig. 2b [23, 24]. When incident EMW strikes the surface of MAMs, they will be reflected, absorbed, or transmitted. MAMs with excellent performance should meet two necessary conditions: first, allow EMW to enter the material as much as possible, that is, appropriate impedance matching; second, the EMW entering the material should be dissipated as much as possible, that is, the so-called attenuation characteristics. How to further improve the performance of MAMs on the original basis is a topic that can never be avoided [25, 26]. At this time, BMAMs have entered the stage of history. Table 1 shows the main application function models of bionics at present. With the continuous improvement in engineering requirements and the continuous development of science and technology, its application will continue to expand [27].

**Table 1:** Correspondence between the form and function of the basic order of biological system [31–42]

Bionic object	Functional primitives and fictional features	Main functional characteristics	References
Plumage	Photonic crystal	Reduced air resistance/schemochrome	[31]
Plant vein	Geometric parting structure	Nutrient delivery/mechanical property	[32]
Polar bear hair	Hollow porous	Keep warm	[33]
Gecko’s foot	Sucker	Adsorption adhesion/dynamic adsorption–desorption	[34]
Lobster shell	Multilayer stacked spiral structure	Toughened	[35]
Spider silk	Spiral and spindle	Toughened/hydrophobic	[36]
Chameleon	photonic crystal	Structural color/dynamic camouflage	[37]
Mollusk	Photonic crystal	Structural color/dynamic camouflage	[38]
Butterfly wings	Scales and grid hole structure	Structural color/hydrophobic heat dissipation	[39]
Shell nacre	Multilayer stack structure	Structural color/toughened	[40]
Moth’s compound eye	Hexagonal subwavelength array	Anti-reflection/water delivery	[41]
Scarab	Helical chiral structure	Rotation/structural color	[42]

The design, controllable preparation, and structural property characterization of bionanomaterials represent cutting-edge fields in materials science and point to active research directions within the discipline [15]. Intelligent microstructure design and judicious composition selection are proven avenues for constructing high-performance MAMs [43]. The emergence of bio-nanofabrication offers a novel strategy for achieving this goal. Nature has evolved intricate and sophisticated microstructures in organisms to exhibit exceptional electromagnetic response behaviors [44]. As depicted in Fig. 2c, the functional primitives and their sequences found on various biological surfaces are the result of billions of years of evolution under the principle of “natural selection and survival of the fittest.” These primitives have adapted to their respective environments, resulting in optimized properties and models. When integrating bionics with MAMs, as shown in Fig. 2d, the biological models inspire novel primitive ordering patterns for absorbing materials, potentially leading to the exploration of new loss mechanisms and breakthroughs in performance. It is true that natural absorbing mechanisms and biological models operate within distinct wavebands compared to artificial MAMs [28, 29]. However, this does not preclude the application of bionics principles to MAMs design. Rather, it necessitates the strategic adjustment of the scale of bionic absorbing elements to match the desired electromagnetic wavelength range. As illustrated in Fig. 2e, the interaction between materials and EMWs is intimately tied to the proportional relationship between their dimensions and the wavelength of the incident radiation. Therefore, in the design of bio-inspired MAMs, the scaling principle must be carefully considered to ensure optimal performance across the target waveband. Moreover, it is important to note that while some bio-inspired designs may mimic the nanoscale structures found in nature and apply them to the millimeter or larger scales of MAMs, others may conversely scale down macroscopic biological features to the nanoscale. The choice of scaling approach depends on the specific application and desired electromagnetic properties of the MAMs [30].

EMWAMs find many applications in our daily lives due to their ability to manipulate and absorb electromagnetic waves [45, 46]. These materials are essential for a variety of industries and technologies, especially those related to the manipulative absorption targets of electromagnetic waves. One major application category includes the use of EMWA materials in military and defense systems to reduce radar detectability and enhance the stealth capabilities of military vehicles and aircraft [45]. Another important category is in the field of electronic communications, where EMWA materials are used to minimize electromagnetic interference (EMI) and improve signal clarity [47]. In addition, EMWA materials have applications in the medical industry, where they are used in MRI machines to absorb and manipulate electromagnetic waves to produce detailed images of the human body [48]. They are also used in consumer electronics such as smartphones and laptops to minimize EMI and protect users from potential health risks. In addition, EMWA materials are increasingly being used in the automotive industry to reduce electromagnetic radiation emissions and improve vehicle safety and efficiency [49]. By categorizing the applications of EMWA materials into these different categories, we can get a clearer picture of their various uses and their relevance to electromagnetic wave manipulation absorption targets.

In this review, the research progress of BMAMs, including preparation methods, bionic principle, absorbing mechanism and simulation, and its future development trend is discussed. At present, the bionic method has been applied to MAMs. If the bionic order principle is applied to MAMs on the basis of in-depth analysis principle and optimization model, it is expected to further improve the absorption performance, and at the same time, new absorption mechanism may be found, which will provide a basis for the theoretical improvement in MAMs.

## Bionic Structure Control of MAMs

After hundreds of millions of years of evolution, the structure and function of organisms have reached a nearly perfect level, and learning from nature has gradually become an important way to develop new materials. Using bionic design principles can provide us with new research ideas to solve some engineering and technical problems [50, 51].

**Fig. 3:**
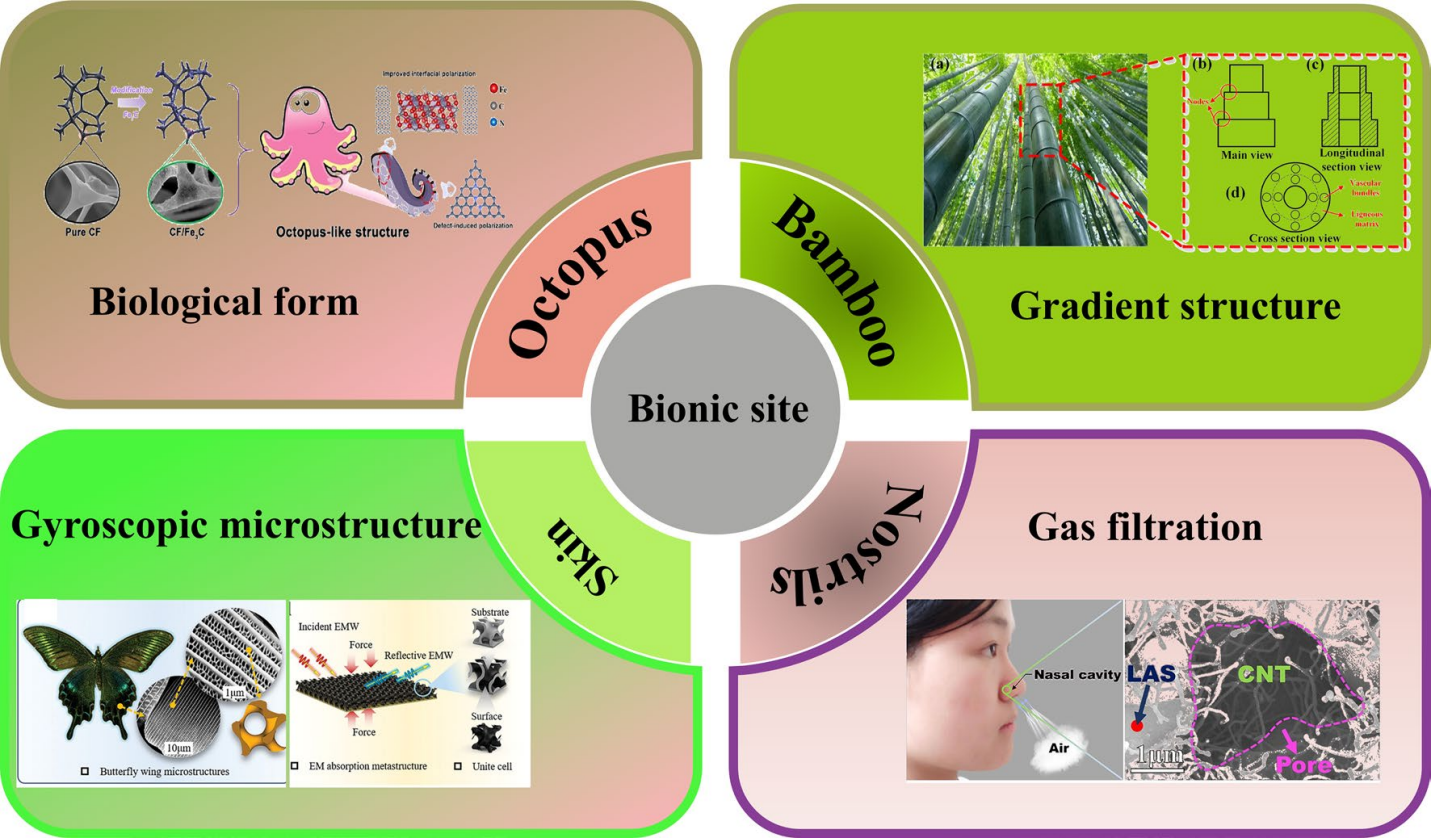
Examples of functional bionics [52–55]

**Fig. 4:**
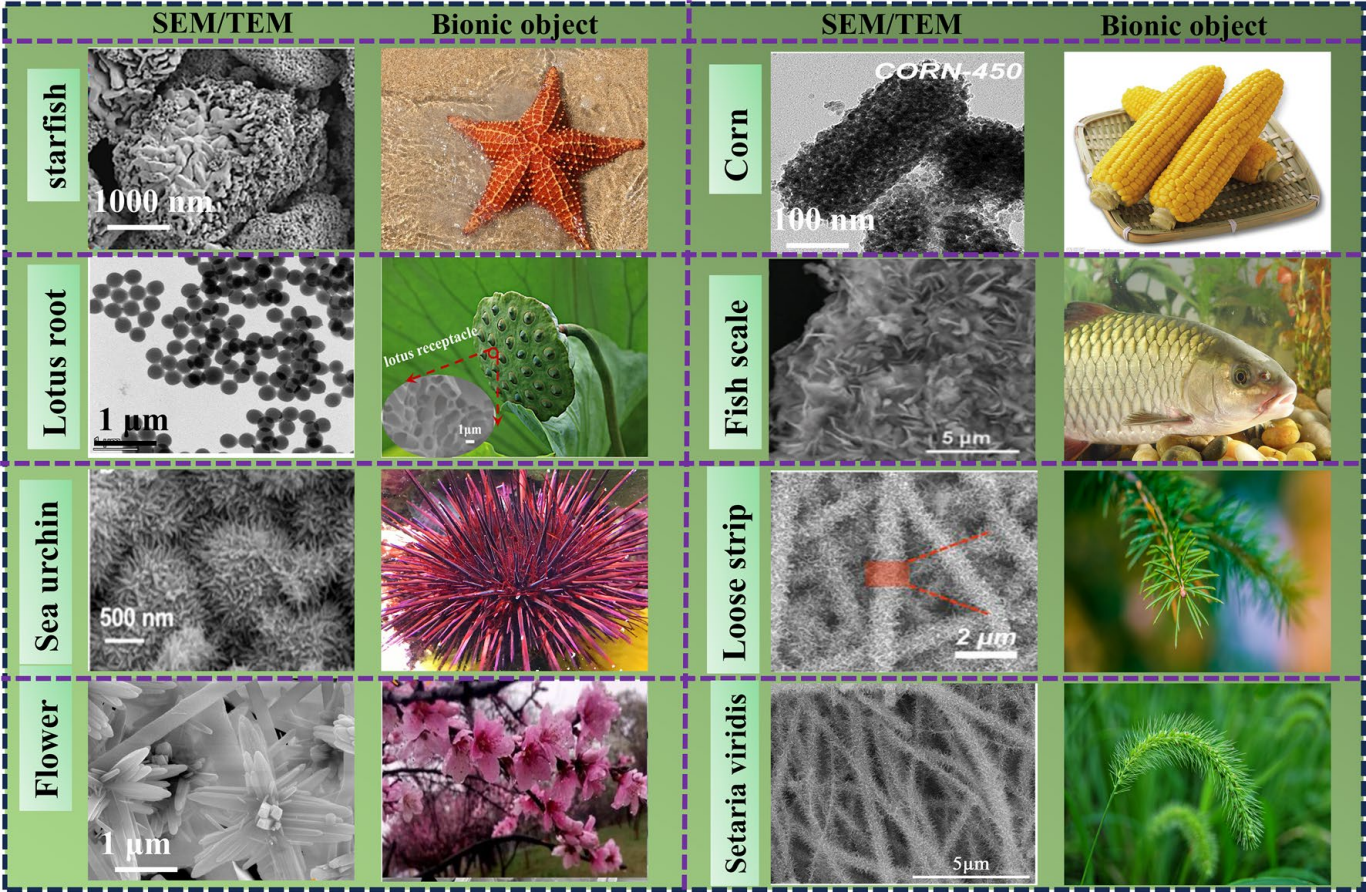
Examples of structural bionics [7, 25, 56–61]

How to combine the innate function or structure of an organism with microwave absorption is a difficult problem. Figure 3 introduces examples of functional bionics, for example, the unit structure of bamboo can be derived into MAMs with multilayer structure, and the filter of human nostrils can be derived into the absorption of EMW. In Fig. 4, structural bionics is introduced. Micro- or macrostructures on the surface of living organisms are used to provide creative inspiration for our experiments, such as the needle-like structure of pine branches, the porous structure of lotus leaves, and the divergent structure of sea urchins. The natural advantages of biological materials have brought researchers a wide range of materials.

Different structural designs will also achieve different MA conditions of materials. Previous studies have studied various structures, such as core–shell [62], cube [63], chain [64], and flower [65]. Because the microstructure is complex, fine, and orderly, people have been exploring various bionic materials that imitate the microstructure of natural materials. This chapter is conceived from two aspects: the selection of bionic components and the design of microstructure, and summarizes the biological model, electromagnetic regulation mechanism, and EMW absorption mechanism of bionic absorbing materials in recent years [66, 67].

### Animal BMAMs

In recent years, the progress of nanotechnology has greatly promoted the research of BMAMs for marine life. Inspired by the excellent stealth ability of marine organisms such as eels and octopuses, researchers have successfully prepared a variety of MAMs with similar structures [68]. By imitating the photosensitive mechanism of marine organisms, these materials construct nano-arrays on the surface of the materials to form bionic microstructures, thus achieving efficient absorption of EMW. These biomimetic marine MAMs can not only significantly reduce the reflected signal in radar and infrared detection, but also improve the stealth capability of military equipment, and show good corrosion resistance, especially suitable for marine environment. Their advantages include light weight, wide efficient absorption of bands (EAB), good absorption performance, strong weather resistance, anti-aging, moisture resistance, pressure resistance, long-term use, non-toxic, and environmental protection [69]. These advances provide new directions and ideas for the development and application of bionic absorbing materials [70].

**Fig. 5:**
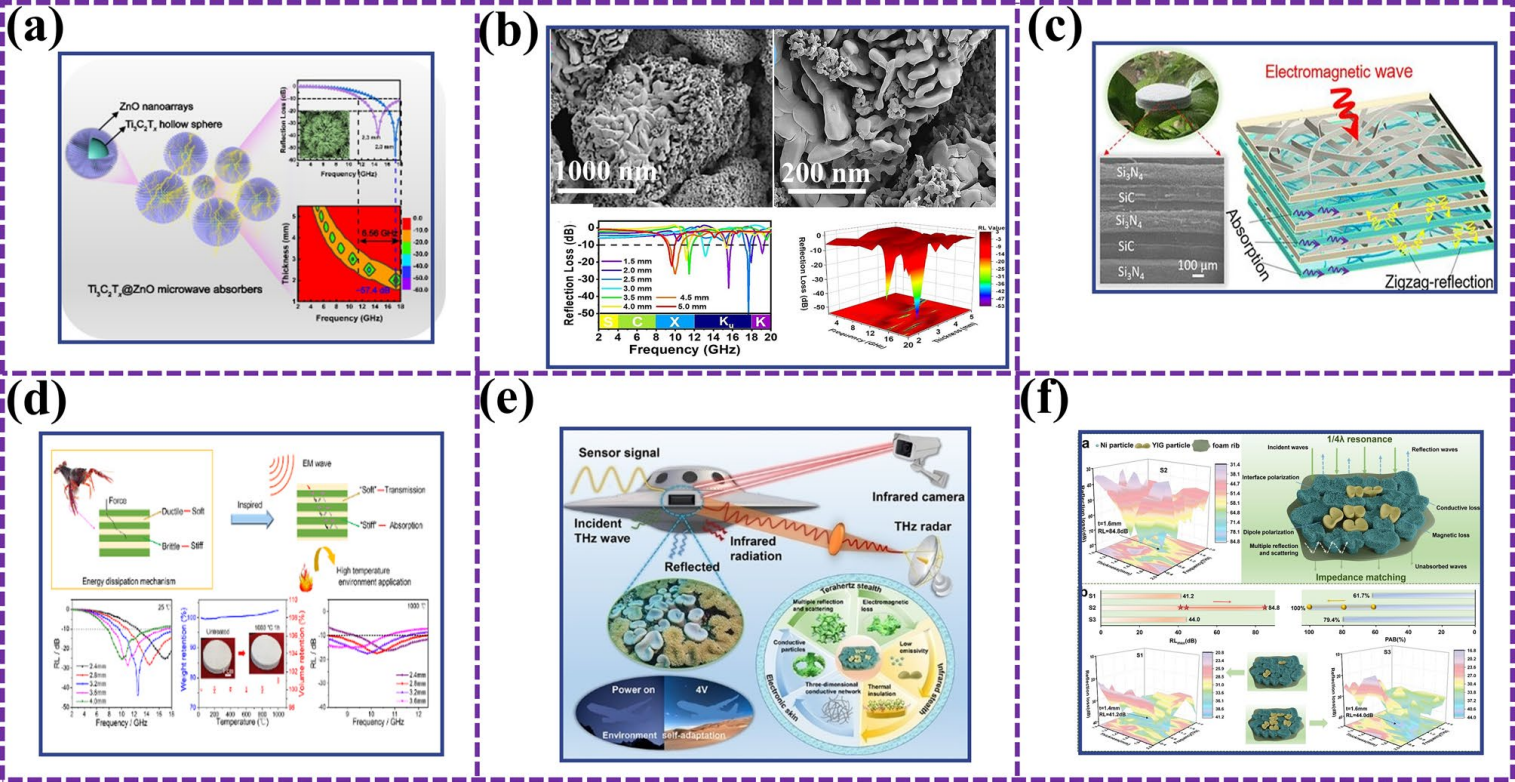
**a** Mechanism and properties of the layered biomimetic absorbing material of sea urchin [71]; **b** SEM images of starfish C/CoNiO_2_ [50]; **c** EMW absorbers modeled on lobster shells for broadband and high-temperature applications [72]; **d** design, preparation, and characterization of multilayer N/C alternate aerogels inspired by impact-resistant lobster shell materials [72]; **e** simulated vitality map of integrated composite material based on intelligent sensing and electromagnetic stealth of natural coral [73]; **f** terahertz and infrared spectrum stealth properties of composite materials and their adaptability [73]

In nature, sea urchin is remarkable for its “seeing” without eyes, which benefits from its unique structure composed of regular spines and spherical photosensitive bodies. Inspired by this, Zhao et al. [71] imitated the structural design of sea urchin and prepared Ti₃C₂Tₓ@ZnO hollow microspheres (as shown in Fig. 5a). This material has excellent performance in the field of microwave absorption, with the minimum of reflection loss (RL_min_) reaching 57.4 dB and EAB reaching 6.56 GHz. This innovation provides a new bionic design idea for MAMs. In addition, the overall symmetry and radial structure of starfish also inspired researchers. Rehman et al. [50] successfully synthesized the heterogeneous sea star-shaped fiber C/CoNiO_2_ (as shown in Fig. 5b), which achieved significantly enhanced electromagnetic attenuation and RL_min_ through local electron polarization and significant magnetic loss. At the thickness of 2.5 mm, the RL_min_ is − 53 dB at 17.65 GHz, while at the thickness of 4.5 mm, the EAB reaches 1.4 GHz (9.3–10.7 GHz). These achievements show the great potential of bionics in the design of MAMs.

Through bionic strategy, Su et al. [72] successfully solved the problems of narrow EAB and limited performance of conventional MAMs in high-temperature environment. Inspired by the fracture dissipation mechanism of soft and hard multilayer structure of lobster shell, they prepared high-performance EMW absorption ceramic aerogels (as shown in Fig. 5c, d), which are composed of multilayer wave-transparent layers SiN and EMW-absorbing layers SiC alternately. This kind of aerogel has a density of 8 mg cm^−2^, an EAB of 8.4 GHz, and RL_min_ is − 45 dB and maintains excellent EMW absorption performance at a high temperature of up to 1000 °C, which indicates a broad application prospect of bionic lobster shell EMW-absorbing materials in high-temperature environment. In the application of equipment, BMAMs also show irreplaceable importance. In order to meet the compatible requirements of intelligent perception and stealth function of equipment in complex and changeable environment, Du et al. [73] designed a coral-like multiscale composite materials. Through bionic structure design, this composite material successfully realizes the combination of intelligent perception and stealth function (Fig. 5e). In terahertz band, the material shows RL_min_ as high as − 84.8 dB, and its EAB covers 0.2–2.0 THz band. In addition, compared with commercial thermal insulation materials, the composite material has lower surface temperature and stable thermal shielding ability. What is even more remarkable is that this material can quickly adjust its temperature like a “temperature-changing dragon” under different power supply voltages, achieving the effect of dynamic infrared stealth (Fig. 5f), providing unprecedented security and interactive means for smart equipment such as electronic skin.

The progress of nanotechnology has promoted the research of BMAMs for marine life. These materials have successfully absorbed EMW efficiently by imitating the unique structure of marine life, such as the photosensitive mechanism or structural characteristics of eels, octopus, sea urchins, and starfish [61]. These BMAMs not only improve the stealth ability of military equipment, but also show good corrosion resistance and environmental adaptability, especially in the marine environment. These advances not only provide a new solution for stealth technology, but also show the great potential and broad application prospect of bionics in the field of materials science. With the continuous progress of science and technology and the expansion of application fields, marine BMAMs are expected to exert greater potential in the future. The multiscale complex fine micro-nanostructure evolved by animals in nature over hundreds of millions of years, especially its electromagnetic response ability, has brought a new perspective for the study of BMAMs [74, 75]. The special structures of insects, butterflies, and chameleons not only inspire the design of new materials for full-band visible light absorption, but also promote the innovation of electromagnetic shielding and stealth technology. For example, the scale structure of butterfly wings inspired researchers to develop absorbing materials with similar characteristics, and its structure was also used to optimize the wing surface design of unmanned aerial vehicle and improve flight performance [76, 77].

**Fig. 6:**
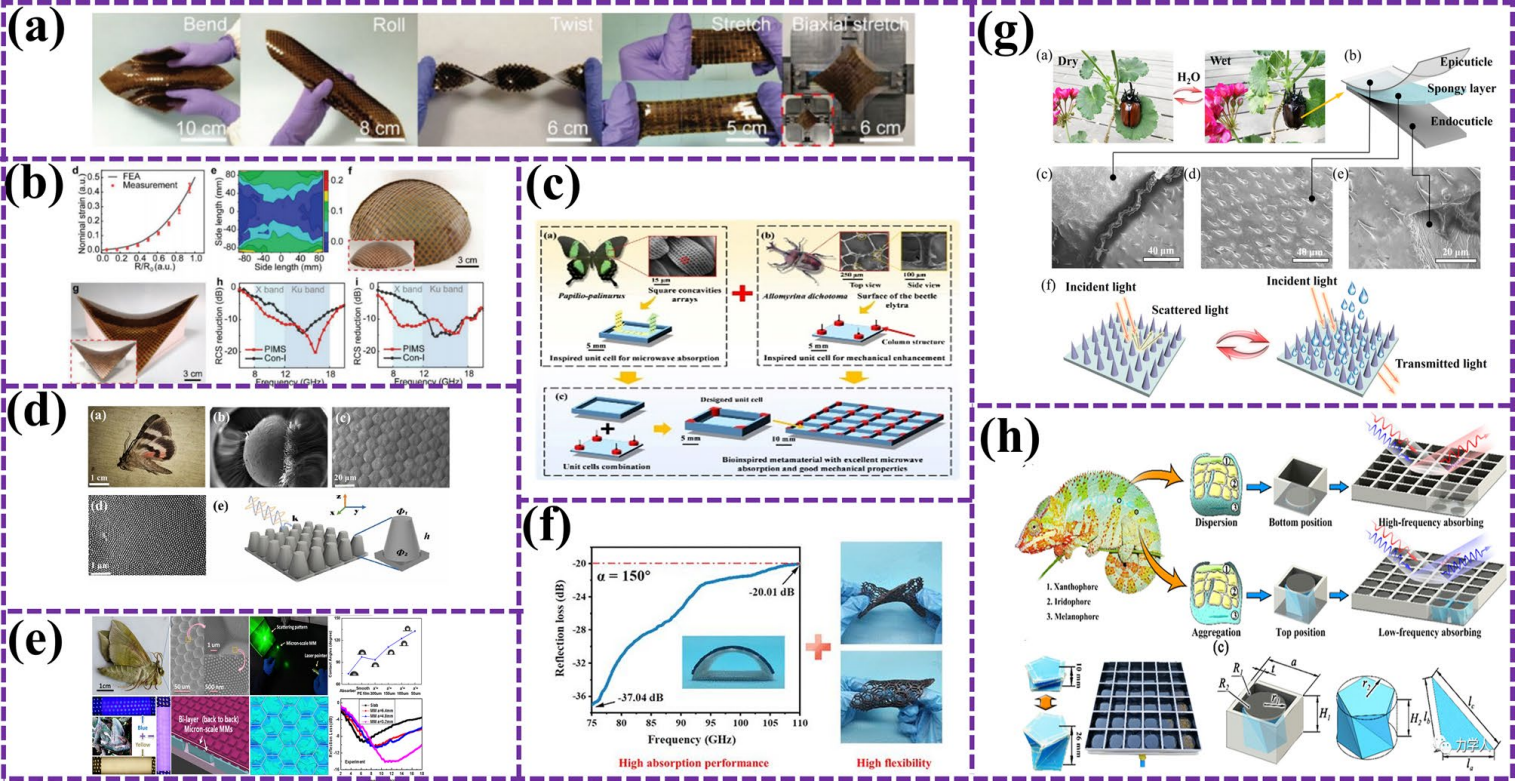
**a** PIMS is easy to bend, roll, twist, stretch, even bidirectional stretch, and has excellent mechanical deformation ability [79]; **b** absorption capacity of devices with nominal strain distribution and deformation into spherical domes and saddle-shaped surfaces [79]; **c** design idea diagram of biomimetic metamaterial with excellent broadband absorption and mechanical properties, and the reflection loss curve comparison between flat plate absorbing materials and biomimetic metamaterial [81]; **d** bionic moth compound eye microstructure as a micro-absorption metamaterial [82]; **e** digital and SEM images of the surface microstructure of real moth eyes, as well as micron-scale ultraviolet spectra and surface water contact angles of various samples [83]; **f** change of color of beetle wing sheath with environmental humidity and schematic diagram of wing sheath structure [84]; **g** schematic diagram of dual-broadband adjustable electromagnetic absorption grid structure design [85]; **h** schematic diagram of the structure design of the simulated chameleon dual-broadband adjustable electromagnetic absorbing grid [52]

However, the lack of extensibility of microwave stealth materials at present limits its application scope. In order to overcome this challenge, scientists took inspiration from pangolin and proposed a new BMAMs based on soft–hard connection strategy [78]. Traditional microwave absorber structures and bulk absorbers have tensile limitations, which limit their application in deformable or special-shaped targets. To overcome these limitations, a conceptually novel soft–rigid connection strategy inspired by the pangolin has been proposed. Chen et al. [79] reasonably designed a stretchable metamaterial pangolin excitation element scale composed of electromagnetic dissipation scale and elastomer. As shown in Fig. 6a, b, the device has strong absorbing ability under 50% tension interference. The maximum radar cross-section (RCS) reduction is 6.3 dB larger than conventional devices, while for saddle-shaped surfaces, the 10 dB RCS reduction increases bandwidth by 83%. In short, this work provides a conceptually novel platform to develop stretchable, non-deployable surface compliant devices. These achievements not only show the great potential of bionics in the design of MAMs, but also provide a new direction for the development of stretchable stealth materials in the future [80].

With the continuous development of intelligent aircraft technology, functional and structural integrated composites have become an important development trend. BMAMs have become the key to achieve EMW stealth and excellent mechanical properties of intelligent aircraft because of their breakthrough in traditional limitations and wide-band EMW-absorbing ability [86]. However, under the limitation of thin thickness, how to achieve broadband impedance matching and good mechanical properties at the same time is still a research difficulty. Inspired by this, Liang et al. [81] innovatively designed a new type of EMW-absorbing and load-bearing integrated metamaterial by combining the bionic design idea and drawing inspiration from the EMW control square groove nanostructure of the angel butterfly wing and the columnar mechanical enhanced array structure of the beetle’s coleopter wing. As shown in Fig. 6c, it not only shows broadband EMW-absorbing characteristics, but also has excellent compressive mechanical properties. The simulation results show that the EAB of the material is 31.7 GHz with a thickness of 6 mm in the frequency range of 2–40 GHz, and the RL_min_ reaches − 46.85 dB at 5.21 GHz. This research not only optimizes the performance of BMAMs, but also provides strong support for the EMW stealth and mechanical properties of intelligent aircraft in the future. Bionics has played an important role in promoting the development of microwave stealth materials and aircraft technology, from stretchable stealth materials inspired by pangolin to functional and structural integrated composites of intelligent aircraft. In the future, with the deepening of research, we are expected to see more applications of high-performance bionic materials in aircraft and other fields [80].

In the research field of BMAMs, although there have been many innovative achievements in EMW absorption and reflection control, the research on broadband absorber in millimeter-wave band of 75–110 GHz is still insufficient, which limits the further development of millimeter-wave technology [87]. In order to fill this gap, He et al. [82] successfully prepared an innovative flexible multi-stage honeycomb absorber based on CIP/MWCNT/ flexible photopolymer resin (FPR) composites, inspired by the nanostructure of ultra-black butterfly scales. Animal eyes are excellent bionic objects. Figure 6d, e shows the BMAMs of two kinds of moth compound eyes, both of which have good EAB. Moth compound eyes are one of the important choices for broadband MAMs. This achievement not only shows excellent EMW absorption performance, but also has excellent flexibility, recoverability, and lightweight characteristics, which provide a new idea for the design of flexible absorber. However, with the diversification of application scenarios, EMW protection materials with single function have been difficult to meet the requirements in complex environments [88]. Therefore, the development of multifunctional EMW protection materials suitable for a wide temperature range has become a new research direction. Duan et al. [83], on this basis, a multifunctional microwave modulator was prepared by vacuum directional impregnation process with beetle wing sheath as bionic object. As shown in Fig. 6e, the modulator not only integrates two EMW protection mechanisms of EMW absorption and radiation deflection, but also achieves an efficient EMW protection effect in a wide temperature range (298–673 K), with a maximum EAB of 5.2 GHz and an optimal EAB protection efficiency of over 97%. In addition, the microwave modulator also has the functions of infrared stealth and real-time monitoring of working temperature, which further improves the stealth ability of materials under various detection methods and the safety threshold when applied in high-temperature environment. This research not only provides experience for multifunctional and intelligent design of high-temperature EMW-absorbing materials, but also shows the broad application prospect of bionics in the field of EMW protection materials.

With the rapid development of radar detection technology, the traditional fixed-band EMW-absorbing structure is inadequate in stealth technology [89]. In order to meet the growing demand for stealth, it is urgent to design an EMW-absorbing structure with active frequency band control ability, especially in realizing lightweight multi-band stealth. However, the current design challenges include narrow frequency band, complex driving, and poor stability [84]. To tackle challenges, researchers sought inspiration from nature. Inspired by chameleon skin’s rapid color change, Lei et al. designed an ultra-wideband EM absorption structure (Fig. 6f). Mimicking chameleon’s pigment–melanocyte interaction, they achieved HF broadband and LF deep band stealth via impedance grating and circular resistor array, complementing for full-frequency EM stealth (Fig. 6h). This showcases bionics’ innovative use in EM absorbers, offering a new research path. Yet, stealth tech faces multi-band adaptability and radar-infrared compatibility hurdles beyond absorber design [85]. Liu et al. inspired by moth compound eyes designed a multi-level metamaterial (Fig. 6g) for broadband microwave absorption, microwave-infrared compatibility, visible light stealth, UV protection, hydrophobicity, and self-cleaning for aircraft anti-icing. This demonstrates bionic design’s potential in stealth tech and offers new ideas. Zhang et al. also achieved in BMAMs, creating high-performance materials inspired by butterfly wings, moth eyes, and scarabs. These flexible, durable, corrosion-resistant materials with broadband and multi-band adaptability, like the Papilio formosana-based material achieving > 90% absorption at 2.18 GHz, reinforce the significance of biomimicry in absorbing materials and stealth tech’s future [52].

When researchers discuss the EMA mechanism, they will inevitably discuss the photosensitive mechanism, which usually refers to the sensitivity of the material to light, that is, the material can undergo certain physical or chemical changes under the condition of light [90]. This mechanism has important application value in the fields of photoelectric conversion, photocatalysis, and light sensor. Photosensitive materials can absorb light energy when exposed to light and convert it into other forms of energy (such as electricity, heat) or trigger chemical reactions [91]. Both photosensitive mechanisms and EMA are involved in energy conversion and absorption. Photosensitive materials absorb light energy and convert it into other forms of energy, while EMA materials absorb electromagnetic wave energy and dissipate it into heat or other forms of energy [92]. Although they involve different types of energy and conversion mechanisms, there are commonalities in energy conversion and absorption. There are some specific examples, photoelectric conversion materials, or light sensors, requiring the material to be both photosensitive and electromagnetic wave absorbent. In this case, there is some overlap between the photosensitive mechanism and the EMA. However, it is important to note that this intersection is not universal, but depends on the specific application scenario and material characteristics. For the design of materials that need to have both of these properties, it is necessary to optimize the composition, structure and application scenarios of the materials [93].

To sum up, bionics has made remarkable progress in the field of electromagnetic absorbing materials. Inspired by natural biological structures such as butterfly wings, pangolin skin, and chameleon skin, researchers have developed new wave-absorbing materials with broadband EMW-absorbing ability, extensibility, versatility, and excellent mechanical properties. These materials not only overcome the limitations of traditional fixed-band electromagnetic absorbing structures, but also show great potential in multi-band adaptability, radar, and infrared stealth compatibility. With the deepening of research, it is expected to see more applications of high-performance bionic materials in aircraft and other fields in the future, further promoting the development of stealth technology. With the deepening of research, there may be more innovative BMAMs based on animal characteristics in the future. Interdisciplinary integration will promote the development of BMAMs for animals and bring more benefits to human society.

### Plant BMAMs

All kinds of plants in the biological world have shown inestimable potential in the field of EMW absorption with their unique fine morphology and rich hole structure [94]. This potential comes not only from their complex biological structures, but also from their excellent performance in energy transmission and transformation. In the process of exploring MAMs, scientists draw inspiration from plants and combine plant BMAMs with biological legacy materials in order to create more efficient and environmentally friendly MAMs [95]. Plant BMAMs simulate the microstructure of plants, and by copying the porous and layered structure in plant leaves or stems, the efficient absorption and conversion of EMW are realized. At the same time, combined with the fine morphology and excellent electron transport characteristics of biological materials, these BMAMs can achieve high-efficiency absorption in a wider frequency band and maintain stable performance in harsh environments such as high temperature and high humidity [57, 96].

**Fig. 7:**
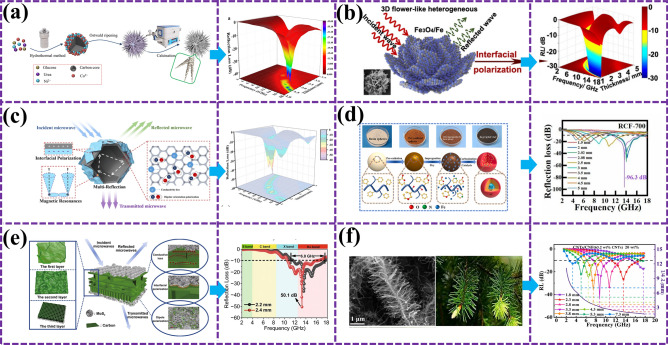
**a** Preparation flow chart and performance diagram of Lamboudan-like dielectric magnetic C@NiCo_2_O_4_ bionic material [97]; **b** microwave absorption mechanism and performance diagram of bionic flower-like Fe_3_O_4_/Fe composite material with adjustable chemical composition [98]; **c** inspired by the electromagnetic response behavior of leaf hopper microstructure, the wave-absorbing mechanism and performance diagram of hollow cavity heterogeneous microstructure BMAMs [99]; **d** wave-absorbing mechanism and performance diagram of bionic pomegranate-like Fe_3_C@graphitic amorphous carbon matrix BMAMs [100]; **e** absorption mechanism and properties of graded layered porous C/MoS_2_ morphologic genetic composite derived from lotus leaf [101]; **f** BMAMs SEM and performance maps inspired by the dense and regular hierarchical structure of pine branches, similar to the hierarchical structure of pine leaves [102]

Biological residual materials show remarkable potential in the field of EMW absorption due to their unique fine morphology, abundant pore structure, and excellent electron transport characteristics. In this regard, Yu et al. [97] provide us with valuable implications. The rambuta-like C@NiCo_2_O_4_ material he successfully prepared has taken an important step in the field of EMW absorption with its excellent microwave attenuation ability. As shown in Fig. 7a, the material has a minimum reflection loss of − 39.0 dB at 17.4 GHz with a specific filler quantity and thickness, and the effective absorption bandwidth covers 4.16 GHz, fully demonstrating its efficient electromagnetic wave absorption performance. Du et al. [98] prepared a bionic flower-like Fe_3_O_4_/Fe composite with adjustable chemical composition using three-dimensional Fe_2_O_3_ as a sacrificial template, breaking through the limitation of Snoek, and the strongest reflection loss of the optimized composite is − 56 dB. At 17.5 GHz, the EAB can cover the frequency range of 12.0–15–5 GHz with a thickness of 1.5 mm (Fig. 7b). However, for researchers pursuing higher performance MAMs, it is indispensable to explore more biological structures in nature for more optimal design inspiration. The porous granular structure of leafhopper reticule is a good example, which is excellent in suppressing EMW reflection and enhancing microwave absorption with its super anti-reflection ability. Inspired by this structure, Wu et al. [99] further developed HCoZnNC@MXene composites with porous hollow structures. This material not only inherits the advantages of leafhopper reticular particles, but also realizes multiple reflections by introducing heterogeneous interfaces to enhance interfacial polarization. As shown in Fig. 7c, the material has a reflection loss of up to − 76.40 dB at 7.50 GHz with a specific packing load and thickness, and its EAB can be modulated flexibly in the range of 3.55–18 GHz.

The difficulty of multi-component regulation and nanostructure design brings challenges to the preparation of high-performance MAMs. Sun et al. [100] prepared a biomimetic pomegranate-like Fe_3_C@graphitic carbon embedded in an amorphous carbon matrix (Fe_3_C@GC/AC). Under the combined action of the unique pomegranate release structure and multiple multi-component loss mechanisms, the reflection loss value can reach a maximum of − 96.3 dB, the matching thickness is 2.08 mm, and the EAB covers 6.38 GHz (Fig. 7d). The in-depth exploration of the bionic structure of multi-component system provides an innovative and effective way for the development of high-performance EMW absorbers. Inspired by nature, Lu et al. [101] prepared a gradient layered porous C/MoS_2_ morphologic genetic composite derived from lotus leaves. After treatment, the biological microstructure of lotus leaves was well preserved. The material has a RL_min_ of − 50.1 dB at a thickness of 2.4 mm and a maximum EAB of 6.0 GHz at a thickness of 2.2 mm (Fig. 7e). A new dielectric model is provided to analyze the electromagnetic properties of non-magnetic material systems. One-dimensional carbon nanomaterials have significant potential in absorption EMW due to their unique properties, but agglomeration, loss, and impedance mismatch have always been challenges in their application. To address these issues, Huang et al. [102] took inspiration from the hierarchy of pine branches in nature and designed a composite based on carbon nanofibers (CNF) derived from bacterial cellulose (BC) and amorphous carbon nanotubes (CNTs). As shown in Fig. 7f, this pine foliage-like hierarchical structure effectively solves the agglomeration problem and significantly enhances the interface polarization ability, achieving a RL of − 68.2 dB and a maximum EAB of 5.5 GHz at a thickness of 2.7 mm, opening up a new road for the research of BMAMs.

The research and development of plant bionic absorbing materials not only promote the technological progress in the field of electromagnetic wave absorption, but also provide a new idea for environmental protection and sustainable development. By mimicking biological structures in nature, we can develop more efficient and environmentally friendly materials that reduce pollution and damage to the environment. In the future, with the continuous progress of science and technology and the deepening of research, we have reason to believe that plant bionic absorbing materials will play an increasingly important role in the field of electromagnetic wave absorption.

### Honeycomb Structure BMAMs

In nature, the honeycomb structure is renowned for its unique mechanical properties and geometric esthetics. Its orderly pore structure is not only lightweight and high strength but also exhibits excellent electromagnetic wave attenuation characteristics [103]. Inspired by this, researchers have begun to explore the application of the honeycomb structure in the design of microwave-absorbing materials, aiming to develop novel, high-performance, and cost-effective absorbing materials by imitating the exquisite construction of nature [104, 105]. The biomimetic honeycomb microwave-absorbing material achieves efficient absorption and conversion of electromagnetic waves through precise control of the material’s microstructure and composition. Its unique three-dimensional honeycomb structure not only increases the material’s specific surface area, providing more scattering and reflection paths for electromagnetic waves, but also dissipates electromagnetic wave energy into heat or other forms of energy through internal resistance and dielectric loss mechanisms [106]. This design approach not only enhances the material’s absorbing performance but also reduces its density and weight, offering the potential for equipment lightweighting. Consequently, research on biomimetic honeycomb microwave-absorbing materials holds significant academic value and presents vast application prospects [107, 108].

**Fig. 8:**
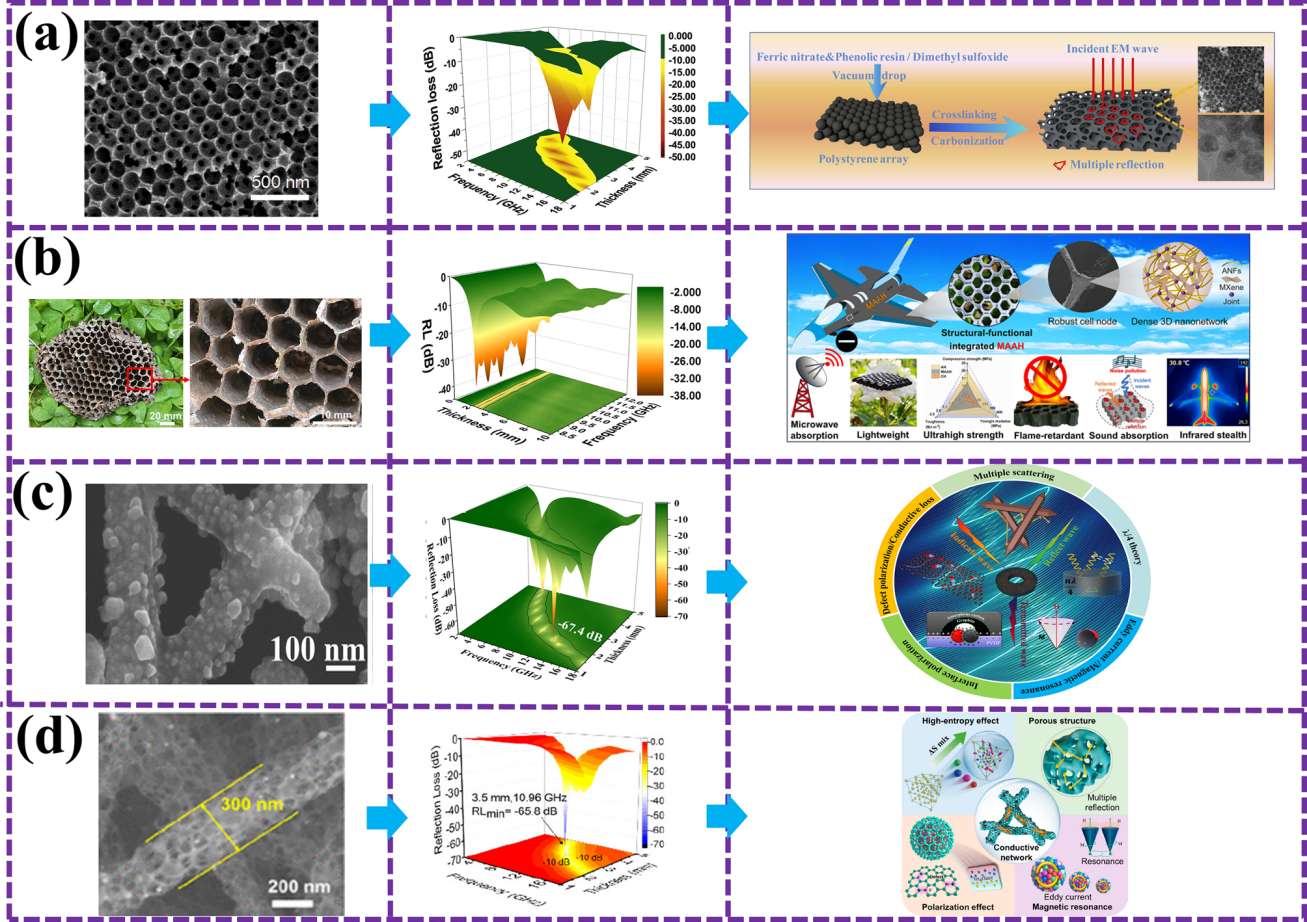
SEM, performance, and schematic diagram of biomimetic honeycomb MAMs. **a** 3D honeycomb nano-Fe_3_O_4_@C composite material [110]; **b** MAAH materials with integrated functional structure [111]; **c** carbon-coated iron matrix composites [112]; **d** cellular structure CNF and HEA [113]

Che et al. [109] designed and fabricated carbon-coated honeycomb MAMs, which boast a lightweight, high stiffness, and broadband performance. Featuring three absorption peaks at 4, 10, and 17 GHz with RL of − 10, − 20, and − 25 dB, respectively, these materials demonstrate remarkable absorption capabilities. Zhang et al. [110] utilized a two-step colloidal templating method to synthesize three-dimensional (3D) honeycomb-like nano-Fe_3_O_4_@C composites and porous carbon structures. As illustrated in Fig. 8a, the Fe_3_O_4_@C composite exhibits a uniform 3D multilayered honeycomb structure with a pore diameter of 100 nm, achieving an optimal RL of 46.4 dB at 9.6 GHz with a thickness of 3.5 mm and an EAB of 5.04 GHz. These enhanced EMWA properties are attributed to the multiple reflections of EMW within the 3D honeycomb structure. Zhang et al. [111] also developed a facile nanofiber-nanosheet assembly strategy to prepare a functional and structurally integrated aramid honeycomb MAMs, as shown in Fig. 8b. Benefiting from the interwoven aramid nanofibers (ANFs) that form integrated, ultra-strong honeycomb nodes and a dense 3D network, microwave absorption aramid honeycomb (MAAH) achieves a RL_min_ of − 38.5 dB at a thickness of merely 1.9 mm, covering almost the entire X-band. Additionally, MAAH exhibits exceptional infrared stealth, sound absorption, and real-time monitoring of structural integrity, indicating its vast potential applications in aerospace, military, and civilian sectors. Lu et al. [112] synthesized MIL-88C(Fe) with various aspect ratios as precursors through controlled oil bath conditions, followed by one-step pyrolysis to obtain carbon-coated iron-based composites. As depicted in Fig. 8c, a symmetric gradient honeycomb structure (SGHS) was constructed using a high-frequency structure simulator (HFSS), achieving an EAB of 14.6 GHz and a RL_min_ of − 59.0 dB. This study provides insights into designing materials and structures with high-efficiency EMWA characteristics. Furthermore, Li et al. [113] successfully fabricated honeycomb-like porous CNF/HEA composites using electrospinning and transient Joule heating methods. As shown in Fig. 8d, HEA nanoparticles are uniformly dispersed within the CNF network, forming a single-phase structure. This composite material achieves a RL_min_ of − 65.8 dB and a EAB of 7.68 GHz with only 2 wt% filler content, setting new records for CNF and HEA absorbers. The study reveals that the synergistic effect between honeycomb-structured CNF and HEA significantly enhances EMWA performance, offering a lightweight and broadband absorption material choice for EMW-absorbing applications.

In summary, biomimetic honeycomb structures have emerged as a promising design paradigm for microwave-absorbing materials due to their unique mechanical properties, lightweight nature, and exceptional electromagnetic wave attenuation capabilities [114, 115]. Researchers have successfully synthesized various honeycomb-structured microwave absorbers through innovative fabrication methods, achieving significant improvements in absorption performance, broadband response, and material efficiency [116–118]. These materials, featuring intricate three-dimensional honeycomb architectures, harness the benefits of increased surface area for enhanced wave scattering and dissipation mechanisms, leading to high absorption capacities and reduced density [119, 120]. Furthermore, the integration of diverse components such as carbon coatings, iron oxides, and carbon nanofibers within the honeycomb framework has further bolstered their electromagnetic properties, enabling efficient energy conversion and dissipation. The development of these advanced materials holds great academic significance and presents broad application prospects, particularly in aerospace, military, and civilian sectors, where lightweight, high-performance microwave-absorbing solutions are highly valued [121].

### Multifunctional Application of BMAMs

The biological model has been almost perfect in the evolution of hundreds of millions of years, and its corresponding functional model has the characteristics of “accurate” regulation [122]. On the one hand, it can avoid the trial and error process in the process of material design and preparation, on the other hand, the living environment of the organism determines the complexity of its body surface microstructure model; its body surface functional primitives have many additional functions besides certain main functions [123]. For example, the moth’s compound eye has a good anti-reflection function besides the main visible light [124]. In addition to being a natural photonic crystal, nacre has a good toughening effect [125, 126]. This provides an effective example for the multi-function of MAMs, which is expected to solve the contradiction between different functions and make MAMs have better practical application value [127].

**Fig. 9:**
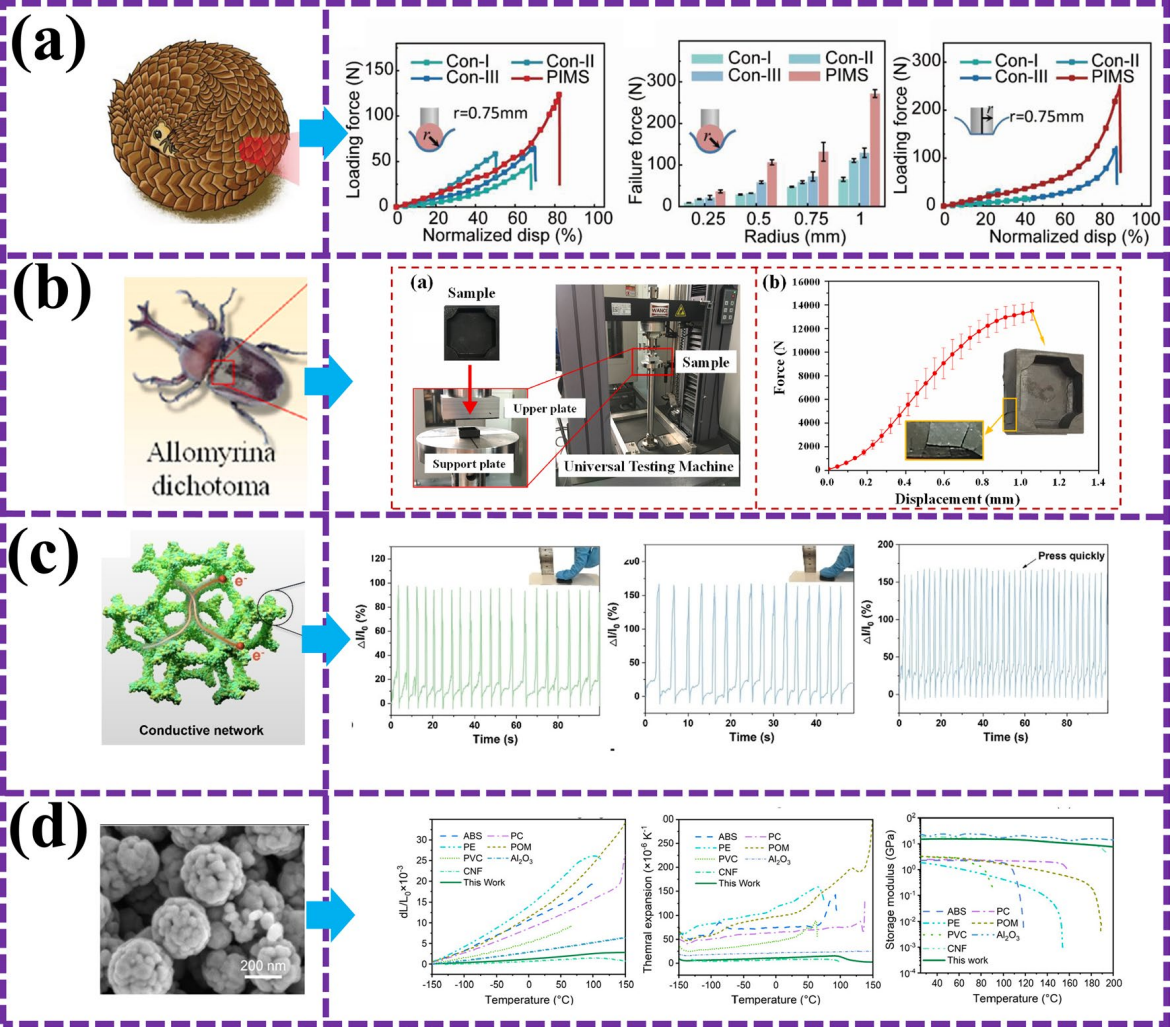
**a** Loading force and normalized indentation displacement curve of pangolin MAMs in spherical indentation test [79]; **b** experimental results of stress resistance of beetle’s elytra MAMs [81]; **c** relation between the relative resistance of biomimetic coral MAMs and the compression strain, the current changes under different strains [70]; **d** bending stress–strain curves of biomimetic cellulose MAMS before and after 20 rapid thermal shock cycles [129]

Chen et al. [79] provide us with a wonderful example. The pangolin-like MAMs not only show excellent EMW-absorbing performance, but also simulate the bite-resistant function of pangolin through out-of-plane press-in test, showing the toughness and practicability of its structure (Fig. 9a). This breakthrough research not only confirmed the effectiveness of bionic design, but also stimulated our interest in further exploration of multifunctional BMAMs. Then, Liang et al. [81] revealed the perfect combination of EMW absorption and mechanical properties of bionic metamaterials. Their optimized biomimetic metamaterials not only have excellent EMW-absorbing properties, but also show excellent compressive mechanical properties. As shown in Fig. 9b, we can clearly see the excellent performance of this material through electrical and mechanical tests. This achievement not only proves the great potential of bionic design in the field of metamaterials, but also provides us with new ideas for the design of multifunctional absorbing materials [128]. Inspired by the sensing mechanism of coral tentacles, Qiu et al. [73] developed a new composite material which integrates intelligent sensing and electromagnetic stealth. This material can not only achieve efficient electromagnetic stealth, but also sense external stimuli through electronic skin function (Fig. 9c), which provides a new solution for equipment independent sensing and intelligent electromagnetic protection. This design idea of integrating multiple functions is exactly what we are pursuing in the field of BMAMs. In addition, Liu et al. [129] found in a comparative study that biomimetic cellulose high-performance MAMs showed significant advantages in thermal properties compared with traditional plastic-based EMA materials. This material not only has a very low coefficient of thermal expansion and stable mechanical properties, but also has better thermal conductivity (Fig. 9d), providing a new option for stealth materials and anti-electromagnetic interference electronic device packaging. This discovery not only broadens the application field of BMAMs, but also provides strong support for our future research and development work [130].

**Fig. 10:**
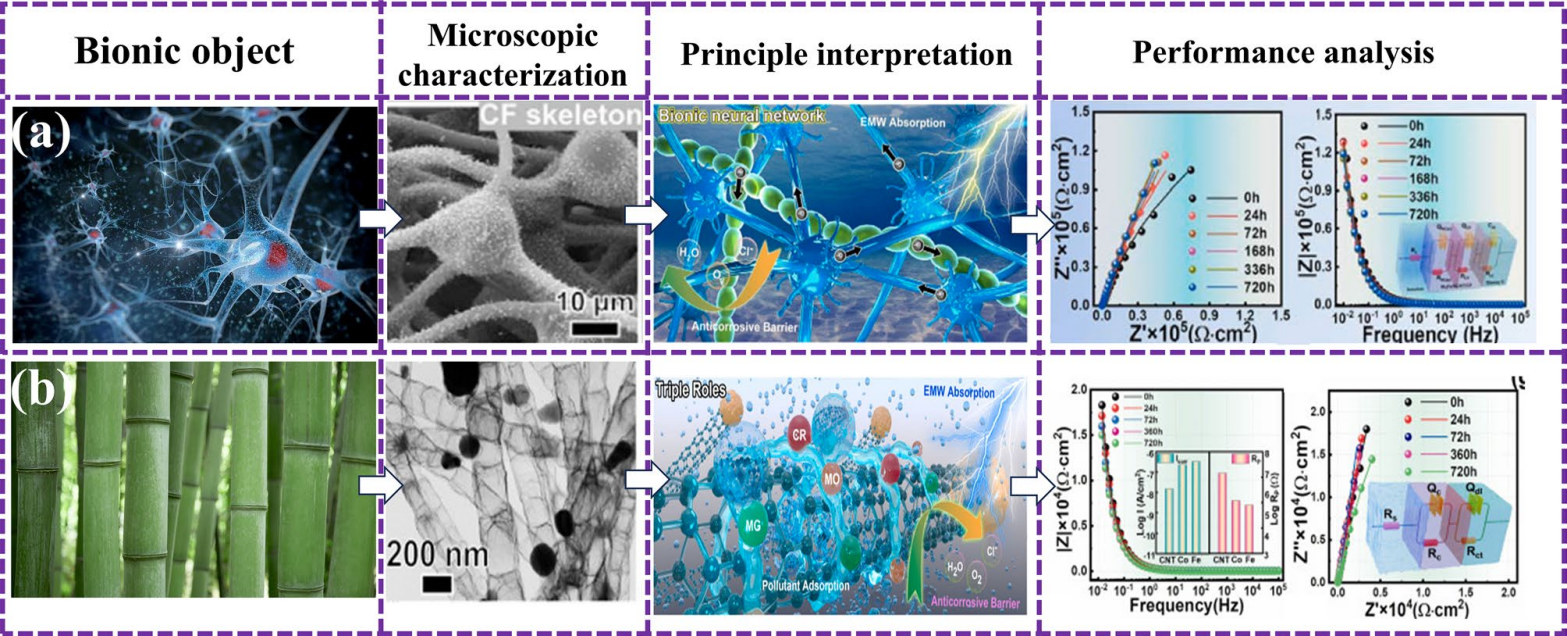
Bionic wave-absorbing materials have two typical cases of microwave absorption and anti-corrosion functions. **a** Bionic neurons [135]; **b** bionic bamboo nodes [134]

BMAMs, with its unique dual functions of anti-corrosion and EMWA, have shown a crucial position in engineering and harsh environment applications. Such materials can not only effectively absorb EMW, reduce electromagnetic interference, but also have excellent corrosion resistance, ensure long-term stable operation under harsh marine conditions such as salt spray and humidity, and provide reliable technical support for marine exploration, communication, and protection [131–133]. To improve the environmental adaptability and survivability of electromagnetic devices in harsh environments, the development of dual-function microwave absorbers faces challenges, as shown in Fig. 10a, Ma et al. [134] innovative design and construction of a 3D layered bionic neural network structure Ni_3_Fe@N-doped CNTs composite material, which realized the integration of EMWA and anti-corrosion. The RL_min_ is − 51.5 dB, and the EAB_max_ is 6.4 GHz at 10 wt%. After 30 days of immersion in corrosive media, the corrosion current density of carbon nanotubes reaches 10^–8 ^A cm^−2^, which has reliable corrosion resistance. The results of this study can provide inspiration for the design of multipurpose microwave materials in complex environments. On this basis, Ma’s team [135] also reported a bionic bamboo-shaped NCNT 3D porous network of EWMA composites coated with magnetic Ni_3_Fe nanocatellites. Only 8 wt% of the 3D network nanocomposite structure can obtain an EAB of up to 6.0 GHz (Fig. 10b). There are long-term stable corrosion resistance in corrosive media (*I*_corr_—10^–8^ A cm^−2^), and excellent adsorption and purification capacity for dye solution (*Q*_*e*_ = 773.6 mg g^−1^). The stepped dielectric regulation of the system can optimize the absorbing performance of the electromagnetic balance, which provides an important idea for the optimal design of MAMs.

We summarized the performance comparison table of BMAMs currently studied. As shown in Table 2, we found that BMAMs indeed have very good performance. We compared EAB, RL_min_, thickness, biomimetic object, and other indicators and found that EAB is one of the best indicators in BMAMs. This also laid the theoretical foundation for our later development of broadband MAMs and broadened the experimental road.

**Table 2:** Comparison of properties of BMAMs

Bionic object	Materials	EAB (GHz)	RL_min_ (dB)	Thickness (mm)	Fabrication method	Key application	References
Moth eye	Graphite powder	13.0	− 35	3.5	Machining	Multi-function stealth	[54]
Moth eye	SCIP/PU	10.2	− 27	2	Metamaterial	Microwave absorption	[136]
Dragonfly eye	FeCoNiSi_8.9_Al_8.9_ C_0.2_ HEA	13.5	− 45	2.8	Hydrothermal	Microwave absorption	[137]
Sea urchin	Fe-Fe_3_O_4_	6.0	− 60	3.9	Sol–gel	Microwave absorption	[61]
Butterfly wing	Gyroid microstructure	38.0	− 49	4.5	Hydrothermal calcination	Microwave absorption	[52]
Spinning top	SiC/Si_3_N_4_	3.5	− 47.6	3.3	3D printing	Microwave absorption	[60]
Corn	Co_2_NiO_4_/Fe_2_O_3_/Fe_3_O_4_	4.4	− 55	5.89	Machining	Microwave absorption	[56]
Octopus	Fe_3_C particle	6.5	− 53.1	2.4	Metamaterial	Microwave absorption	[138]
Fish scale	Graphite/TiC/Ti_3_C_2_	3.5	− 63	2.1	Hydrothermal	Microwave absorption	[94]
Mussel	CNTs-Fe_3_O_4_/Pyrolytic Polydopamine	8.3	− 33	3.5	Sol–gel	Microwave absorption	[139]
Setaria viridis	TiN fiber	10.0	− 47.8	1.11	Hydrothermal calcination	Microwave absorption	[7]
Plants	MXene-based layered aerogel	7.0	− 73.2	3	3D printing	Microwave absorption	[53]
Scarfskin	Graphene film	1.0	− 43.6	1	Machining	Microwave absorption	[59]
Flowers	TiO_2_@SiC/C	5.0	− 45.3	3	Metamaterial	Microwave absorption	[140]
Bamboo	Metamaterials	36.8	− 36	10	Hydrothermal	Microwave absorption	[55]
Human nostrils	Lithium aluminosilicate/CNT	5.9	− 50.5	2.0	Sol–gel	Microwave absorption	[57]
Squirrel branch	ZrO_2_/Co NTs@Ni-doped CNTs	5.4	− 67.9	1.5	Hydrothermal calcination	Microwave absorption	[58]
Chiral spiral carbon fiber	Nepenthes	9.2	− 30.6	4.5	3D printing	Microwave absorption	[141]

Exploring multifunctional BMAMs is an innovative research area. By mimicking natural mechanisms, such as heat dissipation and self-cleaning, BMAMs can enhance microwave absorption efficiency, ensure long-term stability, and reduce maintenance costs. Ahmed Elhassa et al. [142] developed an environmentally friendly composite inspired by ant nests, achieving ultra-thin thickness, wide effective absorption bandwidth, and strong absorption intensity. Duan et al. [83] constructed a metasurface inspired by moth compound eyes, integrating broadband microwave stealth, infrared stealth, visible light camouflage, and self-cleaning functions. Mimicking high-strength structures in nature can improve the mechanical properties and microwave absorption performance of BMAMs. Chen et al. [79] created a stretchable microwave-absorbing material inspired by pangolin scales, demonstrating significant improvements in compressive strength and radar cross-section reduction. In summary, biomimetic microwave-absorbing materials with various excellent properties have broad application prospects.

BMAMs have diverse applications across multiple fields. In sensors, they convert microwaves into other forms of energy, enabling precise detection in structural health monitoring, biomedical imaging, and marine monitoring. For example, a wave measurement system using BMAMs can accurately monitor ocean waves. In flexible electronics, BMAMs’ bendable and stretchable properties make them suitable for flexible displays and solar panels, reducing electromagnetic interference. Their self-healing properties can be used in coatings for automatic repair and continuous electromagnetic property maintenance, with potential in military applications to protect equipment from radar detection. BMAMs also have potential in adaptive camouflage systems, enhancing stealth performance by adjusting microwave absorption. Real-world case studies demonstrate their effectiveness and utility. As technology advances, BMAMs will contribute significantly to more fields, driving scientific and technological progress and social development.

To sum up, from pangolin-like absorbing materials to bionic metamaterials, and then intelligent sensing and electromagnetic stealth integrated composite materials, we have witnessed the continuous progress and development in the field of BMAMs. These studies not only provide us with high-performance solutions for MAMs, but also show us the great potential of bionic design in the design of multifunctional materials. With the continuous progress and innovation of technology, it is believed that BMAMs will show their unique charm and value in more fields in the future.

## Theoretical Breakthrough of BMAMs

### Theoretical Basis of BMAMs

The theoretical basis of BMAMs mainly involves electromagnetic field theory, especially the theoretical system with Maxwell equations as the core [143]. The theory of electromagnetic field reveals the relationship between physical quantities in electromagnetic field and their spatial distribution and time variation. The two core phenomena are that a changing electric field can generate a magnetic field, and a changing magnetic field can generate an electric field [144, 145]. The changing magnetic field and electric field are closely related and inseparable unity, namely electromagnetic field. According to Maxwell’s equation, the electromagnetic field in vacuum has specific relations, including inductive intensity, vacuum dielectric constant, electric field intensity, magnetic induction intensity, vacuum permeability, and magnetic field intensity [146]. When an external electromagnetic field exists, the equilibrium state inside the medium will be broken, resulting in polarization or magnetization of the medium [73, 138, 140, 141, 147, 148].

Impedance matching studies the frequency of EMW and how much EMW can penetrate into the MAMs when the EMW interacts with the material, so reducing the reflection of EMW is the primary condition for the design of MAMs [149]. According to the transmission line theory, the input impedance *Z*_in_ and the free space impedance expression of the absorbing material are, respectively [150]:

1 $$Z_{{{\text{in}}}} = Z_{0} \sqrt {\mu_{r} /\varepsilon_{r} } \tanh \left[ { j\left( {2\pi fd/c} \right)\sqrt {\mu_{r} \varepsilon_{r} } } \right]$$

2 $$Z_{0} = \sqrt {\mu_{0} /\varepsilon_{0} }$$

where ${Z}_{\text{in}}$ is the input impedance of the absorber, ${Z}_{0}$ is the impedance of free space, ${\mu }_{r}$ and ${\varepsilon }_{r}$ are the relative complex permeability and dielectric constant, respectively, $f$ is the microwave frequency, $d$ is the thickness of the absorber, and $c$ is the velocity of the electromagnetic wave in free space[151].

According to the theory of electromagnetic field, when EMW acts on the surface of material, part of it reflects outward and the other part enters the material [59]. The reflectivity at the interface of different materials can be expressed by the reflection coefficient $R$, and the formula is as follows [152]:

3 $$R = \frac{{Z_{{\text{in }}} - Z_{0} }}{{Z_{{\text{in }}} + Z_{0} }}$$

Therefore, when *Z*_in_ = *Z*_*0*_*, R* = 0, which is the ideal state of zero reflection in the design of MAMs. If wave-transmitting materials with excellent insulation properties are selected, zero reflection can be achieved, but such materials have almost no EMW-absorbing ability [138]. Therefore, it is necessary not only to meet the impedance matching, but also to make the MAMs effectively distributed along the transmission path of EMWs, so that EMWs can pass through and be absorbed by the MAMs on the way. It is necessary to adjust the electromagnetic parameters to achieve impedance matching, reduce the reflection of EMW, and strengthen the attenuation and dissipation of EMW entering the material [153].

In order to further discuss the equations of absorbing materials, we should go beyond Maxwell’s equations and discuss metamaterial theory, effective medium theory, and multiscale modeling [154].

Metamaterials are artificial composite structures or composite materials with extraordinary physical properties that natural materials do not have. These properties are derived from artificial special structures, rather than their basic building materials [155]. Metamaterials are composed of subwavelength artificial structural units as basic units, with unit intervals of micrometers. By combining and arranging artificial atoms and artificial molecular units in different ways, metamaterials with various physical properties can be designed and manufactured. The application of metamaterials in BMAMs is mainly reflected in the realization of broadband absorption and multi-functionality. By precisely designing the structure and composition of metamaterials, electromagnetic waves can be effectively absorbed and manipulated to achieve broadband absorption. At the same time, the designability of metamaterials enables BMAMs to have a variety of functions, such as stealth, electromagnetic shielding, and energy harvesting.

Effective medium theory is a theory used to describe the electromagnetic properties of composite materials or mixed media. It is based on the electromagnetic properties of the components in the composite and their interactions to predict the electromagnetic properties of the overall material. Effective medium theory can be used to model and predict the electromagnetic properties of complex biomimitated structures. By considering the electromagnetic properties of different components in BMAMs (such as biomolecules, inorganic materials, etc.) and their interactions, it is possible to predict the electromagnetic response of the overall structure. This is important for designing and optimizing the electromagnetic properties of BMAMs.

Multiscale modeling is a method used to connect physical phenomena at different scales. It allows researchers to predict macroscopic properties from microscopic structural features. In BMAMs, multiscale modeling can be used to understand how microscopic components such as biomolecules and inorganic materials affect the electromagnetic properties of the overall structure. Through multiscale modeling techniques, quantitative relationships between microstructure characteristics and macroscopic properties in BMAMs can be established. This helps to design and optimize the microstructure of BMAMs to achieve specific electromagnetic properties. For example, by adjusting the composition and arrangement of biomolecules and inorganic materials, the broadband absorption and versatility of BMAMs can be optimized.

The theoretical basis of absorbing materials provides a solid guiding significance for the design and development of BMAMs [156]. By deeply understanding the transmission law of EMW in medium, electromagnetic parameters of materials, and absorbing mechanism, we can simulate the unique structure of organisms in nature and design BMAMs with excellent absorbing properties [157]. These materials can not only effectively absorb EMW, but also imitate some functions of organisms, such as bite resistance and perception, so as to realize multi-functional applications [158]. Therefore, the theoretical basis of absorbing materials is an important cornerstone of the research and development of BMAMs, which provides us with innovative ideas and methods [159].

### Broadband Absorption by BMAMs

Theory is the cornerstone of guiding experiments. The mechanism models of MAMs are all traditional models except metamaterials, so it is difficult to make a breakthrough in MAMs, and metamaterials themselves have certain limitations [160]. How to use BMAMs to induce and guide absorbing materials has become the basic condition for its breakthrough [161].

For MAMs, it is not difficult to achieve broadband absorption alone, but if it is limited to deep subwavelength thickness, that is, the thickness is much smaller than the wavelength, it is still difficult to achieve this goal, which is mainly due to the restriction of Plank–Rozanov limit [162, 163]:

4 $$\frac{\Delta \lambda }{d} < \frac{{16\mu_{s} }}{{\left| {\ln \rho_{0} } \right|}}$$

where $\Delta \lambda$ is operating bandwidth, $d$ and ${\mu }_{s}$ are thickness and static permeability of the slab material, respectively, and ${\rho }_{0}$ is reflection coefficient. It can be found that the maximum bandwidth could be increased by increasing ${\mu }_{s}$ under the premise that $d$ is a constant. This formula was deduced by Rozanov in 2000 [162]. The application fields of MAMs, such as absorbing patches in communication equipment and stealth coatings on military equipment, all require materials to reduce the thickness as much as possible and increase the effective absorption bandwidth as much as possible. However, from the above formula, it can be found that the permeability and reflection coefficient of ordinary planar MAMs are determined at the same time after the material of absorbing body is determined, so reducing the thickness and increasing the absorption bandwidth to a certain extent will reach the upper limit of performance and cannot continue [164]. On the other hand, on the premise that the thickness of the absorber is constant, it is necessary to increase the permeability of the absorber or decrease the reflection coefficient to broaden the EAB. However, the quantum properties of magnetic properties are far more difficult to control than electrical properties, and it is still very difficult to control the intrinsic permeability of materials so far [165, 166]. Therefore, it is a feasible method to break through the Plank–Rozanov limit by designing an effective sequence model and improving the permeability or reducing the reflection coefficient with the primitive sequence design [77].

**Fig. 11:**
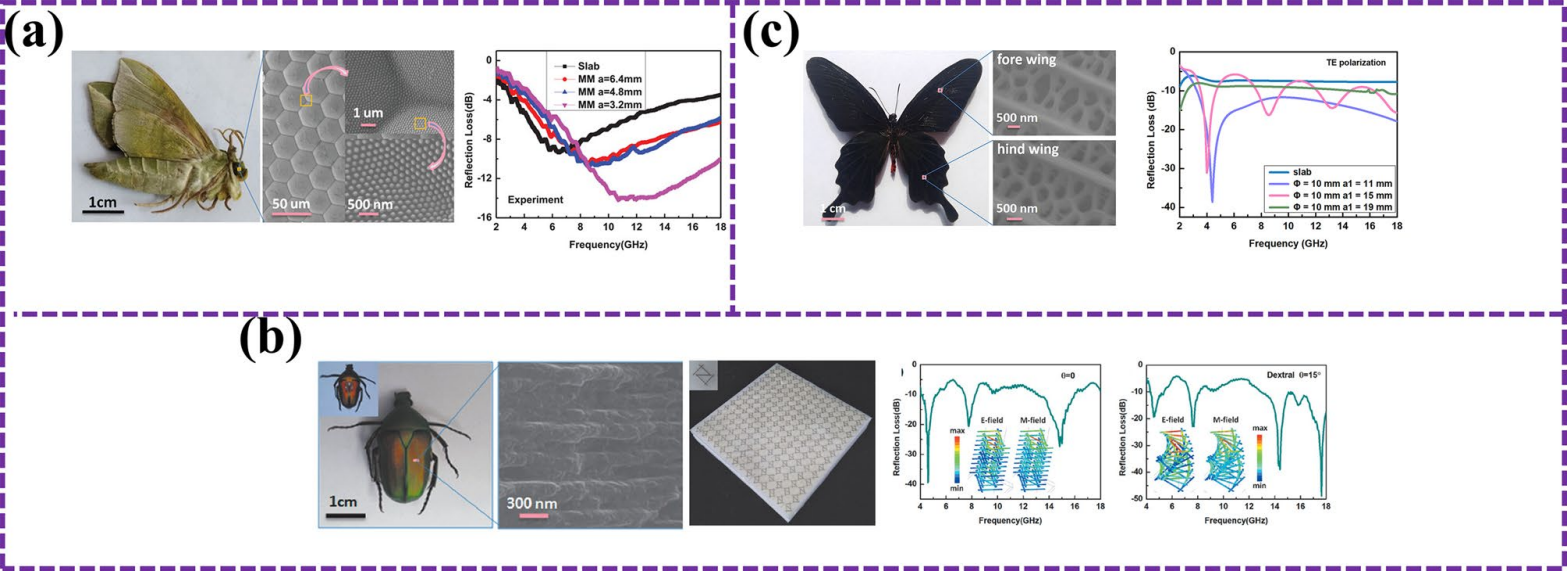
**a** Digital and SEM images of the surface microstructure of real moth eyes and experimental and simulated reflection losses of millimeter-scale metamaterials [167]; **b** RL and corresponding field distribution of bionic chiral material photos and chiral materials with different distortion angles [83]; **c** photographs of *Pachliopta aristolochiae* and corresponding SEM images, RL of bionic specimen and plate specimen with different aperture and period spacing [168]

Bionic design of MAMs can well solve this problem. Inspired by the ultraviolet (UV) model on the surface of moth eyes, Zhang et al. [83] prepared BMAMs covering the entire X and Ku bands (8.04–17.88 GHz) at a deep subwavelength thickness (1 mm) (Fig. 11a). The mechanism explored by simulation models is the discovery of topological effects in biological structures. This discovery points out a way to overcome the physical limitations of mm by using natural models and has broad application prospects in new photonic materials. Duan et al. [167] have designed and prepared spin-oriented metamaterials inspired by the chiral structure of gem beetles, with EAB covering the entire 4–18 GHz frequency range (Fig. 11b), which hold great promise for functionally integrated materials that facilitate stealth technology, counter-terrorism, encryption, sensing, and photon detection. Huang et al. [168] inspired by the absorption model of Primrose proposed a method to achieve ideal stealth with synthetic concept (Fig. 11c). Through the synergistic effect of microwave melanin and bionic superstructure, the bionic metamaterial prepared can effectively absorb radar stealth in the range of 2–18 GHZ and has flexibility and impact resistance at sub-zero temperatures. It can be generalized to many metamaterials and a wide range of absorption.

Based on bionics design as a breakthrough, the wave-absorbing material is combined with biological model, and the biological model with electromagnetic wave absorption in nature is extracted by taking advantage of the evolution of natural organisms to be perfect for hundreds of millions of years. The wave-absorbing material is prepared into bionic primitive and arranged in order, and the bionic primitive is designed as subwavelength scale, that is, the characteristic size of the primitive is equivalent to or smaller than the working wavelength [169]. This scale feature has been proved to have a good anti-reflection effect of electromagnetic wave, so it is suitable to be used in absorbing materials. Breaking through the performance limitations of traditional absorbing materials, the material has broadband absorption performance at deep subwavelength thickness and reveals the microscopic mechanism of bionic materials to achieve broadband absorption, while having multi-frequency and multi-functional adaptability [170, 171].

### Simulation of BMAMs

The simulation of BMAMs is based on electromagnetic field theory, and the propagation, reflection, and absorption of EMWs in materials are accurately simulated by calculation software to evaluate and optimize the absorbing performance of materials [172]. This method can simulate the unique structure of organisms in nature, so as to design bionic materials with excellent EMW-absorbing performance and provide innovative solutions for electronic countermeasures, stealth technology, and other fields [173].

In the field of biomimetic materials and specifically BMAMs, simulation plays a crucial role in understanding and optimizing their performance. Several commonly used simulation software packages, such as COMSOL multiphysics, HFSS (high-frequency structure simulator), and CST (computer simulation technology), are employed to model and analyze these materials.

COMSOL is a powerful multiphysics simulation software that enables modeling of complex physical phenomena. In the context of BMAMs, COMSOL can be used to simulate electromagnetic wave interactions with the material, as well as to analyze the mechanical properties and structural integrity. It offers a wide range of physics modules, including electromagnetics, structural mechanics, and heat transfer, which can be combined to simulate multiphysics interactions. HFSS is a specialized electromagnetic simulation software used primarily for the design and analysis of microwave circuits and antennas. It is well suited for simulating the electromagnetic properties of BMAMs, such as reflection loss, absorption coefficient, and effective bandwidth. HFSS provides accurate predictions of electromagnetic performance and can be used to optimize the design of BMAMs for specific applications. CST is another leading electromagnetic simulation software that offers a range of tools for modeling and analyzing microwave and millimeter-wave components, antennas, and systems. CST can be used to simulate the scattering, absorption, and transmission of electromagnetic waves through BMAMs. It provides powerful tools for visualizing electromagnetic fields and currents, as well as for performing parametric studies and optimization.

In modeling BMAMs, several specific techniques are commonly employed. Finite element method (FEM): This numerical method is widely used in simulation software to solve partial differential equations that describe physical phenomena. In the context of BMAMs, FEM can be used to model the electromagnetic and mechanical properties of the material by discretizing the continuous domain into smaller, manageable elements. Finite difference time domain (FDTD): FDTD is a computational electromagnetics method used to solve Maxwell’s equations in the time domain. It is particularly well suited for simulating transient electromagnetic phenomena, such as the propagation and scattering of electromagnetic waves through BMAMs. Transmission line matrix (TLM): TLM is a method for modeling and analyzing microwave circuits and systems. It can be used to simulate the electromagnetic properties of BMAMs in a microwave environment, including reflection, transmission, and absorption.

Despite the advancements in simulation software and techniques, simulating complex biomimetic structures such as BMAMs remains challenging. Several limitations and challenges are associated with this process. Biomimetic structures often exhibit complex geometric shapes and material compositions, which can lead to high computational complexity. This can make it difficult to simulate these structures accurately and efficiently. Accurately modeling the electromagnetic and mechanical properties of biomimetic materials can be challenging. These properties can vary significantly depending on the composition, structure, and processing conditions of the material. Biomimetic structures often involve multiple physical phenomena, such as electromagnetism, mechanics, and thermodynamics. Simulating these interactions accurately requires a comprehensive understanding of the underlying physics and the ability to combine multiple simulation techniques. Simulating complex biomimetic structures is only as good as the experimental data used to validate the models. Obtaining accurate and reliable experimental data for these structures can be difficult and time-consuming.

Simulation software programs such as COMSOL, HFSS, and CST, along with specific techniques like FEM, FDTD, and TLM play a crucial role in modeling and analyzing BMAMs. However, simulating complex biomimetic structures remains challenging due to computational complexity, material properties, multiphysics interactions, and the need for experimental validation. Despite these challenges, ongoing advancements in simulation technology and techniques are likely to continue to drive progress in the field of BMAMs and their applications in stealth technology and other areas.

**Fig. 12:**
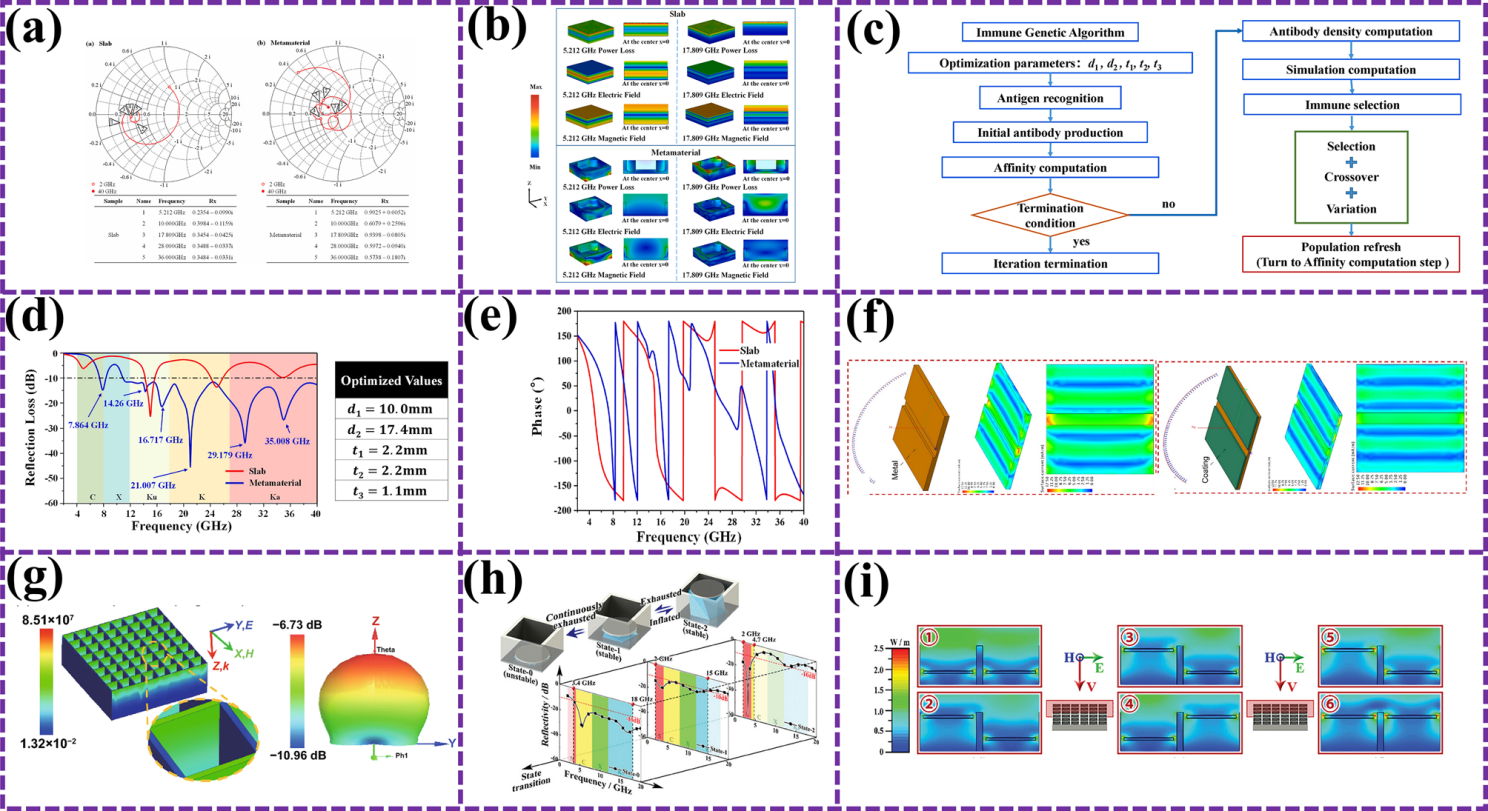
**a** Smith plots of plates and biomimetic metamaterials, where R_x_ is the normalized impedance of the samples [81]; **b** simulating the power losses, electric and magnetic fields of flat plates and biomimetic metamaterials at peak frequencies [174]; **c** immune genetic algorithm flow chart [174]; **d** comparison of reflection loss between the plate and the bionic metamaterial with optimized size under incident TE-polarized microwave [175]; **e** phase and group delay comparison of plate and bioexcited metamaterials [176]; **f** metal plate models and corresponding color diagrams simulating current distribution [177]; **g** maximum electromagnetic protection efficiency (MEPE), EMW volume loss density, and three-dimensional far-field radiation pattern of the sample [178]; **h** electric field cloud image and surface power loss density cloud image simulation results considering the actual state [178]; **i** comparison of experimental and simulation results in the third derivative state [178]

In Liang et al. [81], in order to further reveal the EMW absorption mechanism of biomimetic metamaterials, the normalized impedance diagram in Fig. 12a was analyzed. Compared with flat absorbing materials, biomimetic metamaterials show significant improvement in impedance matching; especially at 5.212 GHz, its impedance reaches 0.9925 + 0.0052*i*, which is very close to free space, indicating that biomimetic design effectively improves the impedance matching problem between materials and free space. Figure 12b further explores the power loss at the peak frequency and the distribution of electric and magnetic fields, clearly showing that magnetic loss is the dominant form of EMW loss of the biomimetic metamaterial. This design not only improves the electromagnetic energy loss intensity in the resonant peak frequency range, but also realizes the perfect combination of wide-band EMW absorption and high absorption intensity. In order to further optimize its structural parameters to achieve broader frequency domain coverage and more efficient EMW absorption, Liang et al. [174] adopt immune genetic algorithm. As shown in Fig. 12c, a multi-objective optimization algorithm for the structural parameters of the wideband EMW-absorbing metamaterials was developed, aiming to achieve impedance matching optimization design in the wideband domain under thin thickness conditions. The implementation effect of this optimization strategy is remarkable. Figure 12d, e shows the simulation results of the reflection loss value of the designed metamaterial against the normal incident electromagnetic wave in the frequency range of 2–40 GHz. Impressively, at a thickness of only 5.5 mm, the optimized biomimetic metamaterial achieves an EAB of up to 29.1 GHz. Compared with the flat absorbing structure, the electromagnetic performance of this design has been greatly improved [175, 176].

After verifying the excellent performance of BMAMs on flat structures, Li et al. further explore their application potential in complex structures. In order to comprehensively evaluate the absorption and suppression effect of such absorbent composites on EMW, Li et al. [177] simulated the effect of RCS reduction on BMAMs. As shown in Fig. 12f, BMAMs show strong EMW control ability, which effectively inhibits the reflected waves, diffraction waves, and traveling waves generated by strong scattering structures such as gaps and dihedrons, significantly reduces the RCS of the original structure, and provides a new solution for stealth technology in radar band [16]. However, relying on absorbing coatings alone may not meet the needs of all application scenarios. Duan et al. [178] further studied the CIP/C-wood bionic microwave modulator, which not only has excellent EMW absorption capability, but also achieves deflection of reflected waves. As shown in Fig. 12g, the structural design of CIP/C-wood spacing arrangement breaks the translation invariance of EMW characteristics, causing multiple phase mutations in the range of 8.2–18.0 GHz and realizing large angle deflection of EMW. This discovery provides new ideas for designing more flexible and efficient MAMs. In order to further expand the application range of MAMs, the application of adjustable origami structure in EMW stealth technology was explored. Optimization design of structural parameters by particle swarm optimization algorithm, Duan et al. [178] successfully realized the complementary effect of high and low absorption frequency bands. As shown in Fig. 12h, BMAMs have an EAB of 3.4–18 GHz in the fully contracted state. This complementary design enables the structure to achieve EMW stealth in the full-frequency band range of 2–18 GHz. In addition, Duan et al. [178] also conducted an in-depth analysis of the EMW absorption mechanism of the grid structure (Fig. 12i) and found that the precise control of each cell state can be achieved through the digital regulation strategy, so as to achieve continuous changes in the EMW absorption effect [179].

In summary, the simulation of bionic absorbing materials has the advantages of high efficiency, accuracy, flexibility, cost-effectiveness and easy integration and optimization. These advantages make simulation technology play an important role in the development of absorbing materials, helping to improve product design quality, reduce cost risk, and provide more design freedom and innovation space.

### Bionic Gradient Design

In the long-term process of natural selection and evolution of organisms in nature, the organizational structure and performance of their constituent materials have been continuously optimized and improved, so that simple minerals and organic materials such as raw materials can well meet the complex mechanical and functional needs, so that organisms can achieve the best adaptation to their living environment [24]. Nature is a good teacher of man. The excellent properties of natural biomaterials can provide beneficial enlightenment for the optimal design of artificial materials, especially for the development of high-performance biomimetic materials [180]. Among them, functional gradient design is one of the basic performance optimization strategies commonly used in biomaterials. It is important to reveal the gradient design criteria in nature and the corresponding performance optimization mechanism for guiding the design of high-performance biomimetic gradient materials and promoting their application [181]. The gradient design of BMAMs draws on the structural characteristics of organisms in nature and realizes the efficient absorption and suppression of EMW by carefully constructing the hierarchical distribution of different materials or structures [182]. This design allows the material to undergo continuous or gradient changes in physical and chemical properties along a certain direction to adapt to different environments and achieve specific functions [183].

**Fig. 13:**
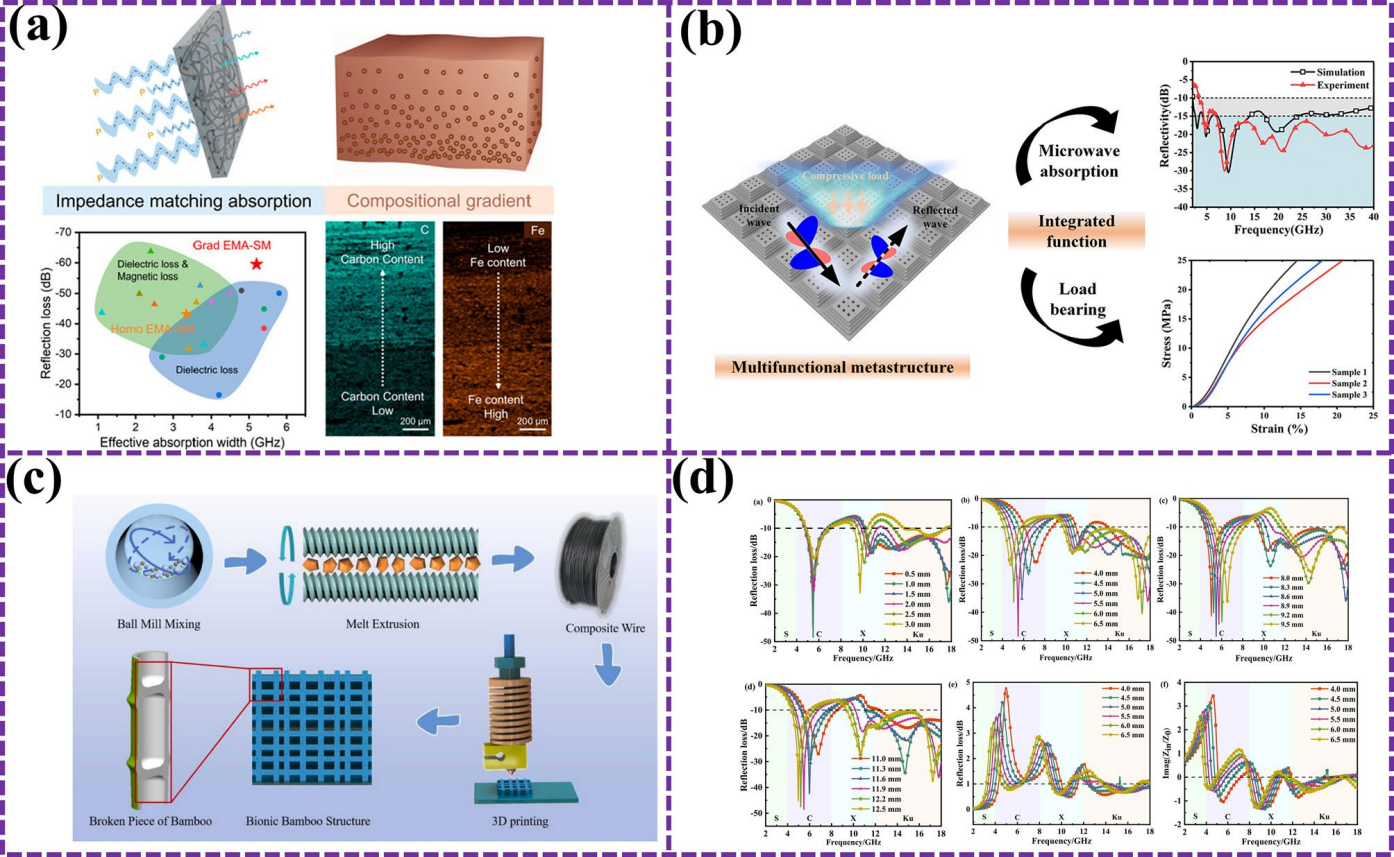
**a** Impedance matching diagram and composition gradient diagram of biomimetic cellulose microwave absorption material, and performance comparison diagram are compared with Fe/C gradient distribution diagram [129]; **b** bamboo-inspired metastructure diagram and microwave absorption and mechanical properties diagram [55]; **c** CB/PLA-TPU 3D printing bionic bamboo structure preparation process diagram [87]; **d** optimization of the geometric parameters of the cell of the microwave absorber and the performance curve [184]

Yu et al. [129] reported the development of a CNF-based structural material by biomimetic gradient structure design, using hollow magnetite nanoparticles and phosphorylated CNF as building blocks, while achieving excellent mechanical properties and EMA capabilities. As shown in Fig. 13a, the gradient design principle and characterization diagram enables the bending strength of the structural material to reach 205 MPa, while contributing to high EMW absorption capacity (− 59.5 dB) and wide EAB (5.20 GHz). Absorbing structures that can cope with complex electromagnetic and physical environments at the same time have become an indispensable part of contemporary stealth technology. Liang et al. [55] studied a bamboo composite superstructure with wide-band EMW absorption and mechanical bearing functions by combining gradient structure design. As shown in Fig. 13b, the EAB range from 3.2 to 40 GHz is realized, and the average compressive yield stress is 13.27 MPa. Also from bamboo, Ye et al. [87] Produced a novel double-layer microwave absorber structure (Fig. 13c), namely CB/PLA-TPU composite bionic bamboo joint structure, which achieved an EAB of 3.84 GHz. The RL_min_ at 2.58 mm thickness and 9.2 GHz frequency is − 60.24 dB (Fig. 13d). The broadband absorption of 10.03 GHz and the yield strength of 11.6 MPa are achieved by experimental simulation. The bamboo-based bionic gradient design offers great potential for the engineering application of stealth technology under harsh conditions [184].

These results show that the integrated functional design is helpful to the practical engineering application of stealth technology in harsh service environment. Gradient design not only demonstrates the great potential of BMAMs, but also provides a new strategy for the design and preparation of high-performance MAMs.

## Conclusion and Prospect

### Conclusion

This review delves into the research progress and future application potential of BMAMs, inspired by the remarkable electromagnetic response capabilities of complex morphologies and subtle microstructures found in nature’s organisms. BMAMs achieve high-performance microwave absorption through ingenious design of microstructures and meticulous selection of compositions. Furthermore, the review meticulously analyzes how to draw inspiration from intricate structures found in marine organisms, plants, animals, and non-metallic minerals in nature to design and develop BMAMs with outstanding EMWA properties. Additionally, the review delves into the theoretical foundation of BMAMs, particularly the latest breakthroughs in the field of broadband absorption. By integrating advanced methodologies such as simulation modeling and biomimetic gradient design, the scientific principles underlying the microwave absorption mechanisms of BMAMs are elucidated, providing a solid theoretical foundation for understanding and optimizing their performance.

Biomimetic materials, inspired by butterfly wings, magnetoreceptor proteins, and tree leaves, offer unique advantages such as lightweight strength, broadband EMW absorption, high sensitivity, and biocompatibility. They are environmentally friendly and sustainable. However, they can be complex and costly to produce, and may not meet high-performance requirements or be scalable for large-scale industrial applications. Conventional MAMs, while simpler and more scalable, may lack these performance characteristics and have a larger ecological footprint. The choice between biomimetic and conventional MAMs depends on balancing performance, cost, and environmental considerations for specific applications.

Overall, BMAMs, by mimicking the unique structures of nature, exhibit superior EMWA characteristics, offering significant value as a reference for the development of novel, efficient, and lightweight MAMs. They also hold profound scientific significance in radar stealth material technology, enhancing the survivability and defensive capabilities of weaponry and equipment. Despite remarkable progress in this field, where broadband EMWA capabilities have been achieved through the integration of advanced nanomaterials and nanostructures with unique properties and natural structures, BMAMs still face challenges related to scalability, durability, stability, and cost-effectiveness.

### Prospect

To sum up, while the research on MAMs has made remarkable progress, it still faces a series of theoretical and application challenges. In order to further improve the performance of MAMs and expand their application scope, future research can be explored from the following aspects:

1. Thickness and broadband absorption performance: further reduce the thickness of the material to achieve broadband absorption, break through the Plank–Rozanov limit, and make the absorbing material have excellent performance at deep subwavelength thickness. Focus on developing strategies to break through the Planck–Rozanov limit and achieve significant absorption at deep subwavelength thicknesses. This may involve exploring new material compositions, structures, and surface treatments that enhance absorption efficiency while maintaining low thickness.
2. Topological model and quantitative evaluation: using topological theory or other mathematical and physical models to quantitatively evaluate the EMA absorbing properties of bionic ordered materials, and provide more accurate theoretical guidance for material design. Multilayer chiral elements and near-field coupling: arranging multilayer chiral elements by spiral order enhances the EMW-absorbing performance of periodic ordered materials and explores the form and order characteristics of elements that maintain near-field coupling while reducing the thickness of interlayer. Analyze the potential of applying topological theory to quantitatively evaluate and design BMAMs with improved performance. This could involve developing models that incorporate topological invariants to predict and optimize material behavior under various electromagnetic conditions.
3. Development of new theoretical models: develop new mathematical, physical and bionic models, break through the limitations of traditional theoretical models of MAMs, and further improve the absorbing performance.
4. Multi-band compatibility and functionality: solve the compatibility problem of MAMs with other bands (such as infrared, visible light, etc.), study multi-band adaptability, and develop multifunctional absorbing materials. Investigate how to address the compatibility challenges of BMAMs with other frequency bands, such as infrared and visible light. This research should aim to develop materials that can efficiently absorb microwaves while maintaining transparency or low absorption in other spectral regions.
5. Scaling up the production of BMAMs from laboratory prototypes to industrial scale entails challenges in material consistency, process control, cost-effectiveness, and scalability. Techniques such as 3D printing, bio-templating, and self-assembly offer potential solutions but require further optimization. Additional research and development are necessary to ensure feasibility for large-scale industrial applications.

From the perspective of BMAMs, the realization of effective absorption bands with wider frequency ranges and lower frequencies can be pursued through the exploration of the following avenues:

1. Intensified biomimetic research: delve deeper into the electromagnetic response mechanisms of various biological entities in nature (such as marine organisms, plants, and animals), particularly those capable of efficiently absorbing electromagnetic waves across extremely wide frequency spectra. By uncovering their structural characteristics at both micro- and macro-scales, and their interactions with electromagnetic waves, we can garner inspirations for designing novel BMAMs.
2. Multiscale structural design: incorporate biomimetic principles to design BMAMs featuring multiscale structural architectures. This design approach spans from nanoscale to microscale and beyond, leveraging the synergistic effects between structures of different scales to achieve broader bandwidth microwave absorption.
3. High-performance composite development: explore novel composite material systems that integrate materials with exceptional electromagnetic properties into lightweight, high-strength matrices, producing BMAMs that excel in microwave absorption while meeting practical application requirements. Furthermore, refine the material’s composition, structure, and fabrication processes to enhance its microwave absorption efficiency and stability.
4. Theoretical modeling and simulation: establish and refine theoretical models for BMAMs, leveraging advanced simulation technologies to predict and optimize their microwave absorption capabilities. Through a synergy of theoretical calculations and experimental validation, gain profound insights into the microwave absorption mechanisms of BMAMs, thereby providing a solid scientific basis for material design.
5. Environmental adaptability research: address the environmental adaptability concerns of BMAMs in practical applications, such as the effects of temperature, humidity, and pressure on material performance. By adjusting material formulations and structural designs, enhance the stability and reliability of BMAMs across varying environments, thereby expanding their application domains.

In conclusion, achieving BMAMs with wider and lower absorption frequency bands necessitates a multidisciplinary approach that integrates knowledge and techniques from biomimetics, materials science, physics, and beyond. Through relentless research and innovation, we are confident that more efficient, stable, and environmentally friendly BMAMs will be developed, significantly contributing to advancements in electromagnetic shielding, stealth technology, wireless communications, and other related fields.
